# Abnormal Pyramidal Decussation and Bilateral Projection of the Corticospinal Tract Axons in Mice Lacking the Heparan Sulfate Endosulfatases, Sulf1 and Sulf2

**DOI:** 10.3389/fnmol.2019.00333

**Published:** 2020-01-21

**Authors:** Satoshi Aizawa, Takuya Okada, Kazuko Keino-Masu, Tri Huu Doan, Tadachika Koganezawa, Masahiro Akiyama, Akira Tamaoka, Masayuki Masu

**Affiliations:** ^1^Graduate School of Comprehensive Human Sciences, University of Tsukuba, Tsukuba, Japan; ^2^Department of Molecular Neurobiology, Division of Biomedical Science, Faculty of Medicine, University of Tsukuba, Tsukuba, Japan; ^3^Department of Neurology, Division of Clinical Medicine, Faculty of Medicine, University of Tsukuba, Tsukuba, Japan; ^4^Department of Physiology, Division of Biomedical Science, Faculty of Medicine, University of Tsukuba, Tsukuba, Japan; ^5^Transborder Medical Research Center, Faculty of Medicine, University of Tsukuba, Tsukuba, Japan; ^6^Environmental Biology Laboratory, Faculty of Medicine, University of Tsukuba, Tsukuba, Japan

**Keywords:** heparan sulfate, Sulfatase 1, Sulfatase 2, knockout mouse, corticospinal tract, pyramidal decussation, motor movement, bilateral projection

## Abstract

The corticospinal tract (CST) plays an important role in controlling voluntary movement. Because the CST has a long trajectory throughout the brain toward the spinal cord, many axon guidance molecules are required to navigate the axons correctly during development. Previously, we found that double-knockout (DKO) mouse embryos lacking the heparan sulfate endosulfatases, *Sulf1* and *Sulf2*, showed axon guidance defects of the CST owing to the abnormal accumulation of Slit2 protein on the brain surface. However, postnatal development of the CST, especially the pyramidal decussation and spinal cord projection, could not be assessed because DKO mice on a C57BL/6 background died soon after birth. We recently found that *Sulf1/2* DKO mice on a mixed C57BL/6 and CD-1/ICR background can survive into adulthood and therefore investigated the anatomy and function of the CST in the adult DKO mice. In *Sulf1/2* DKO mice, abnormal dorsal deviation of the CST fibers on the midbrain surface persisted after maturation of the CST. At the pyramidal decussation, some CST fibers located near the midline crossed the midline, whereas others located more laterally extended ipsilaterally. In the spinal cord, the crossed CST fibers descended in the dorsal funiculus on the contralateral side and entered the contralateral gray matter normally, whereas the uncrossed fibers descended in the lateral funiculus on the ipsilateral side and entered the ipsilateral gray matter. As a result, the CST fibers that originated from 1 side of the brain projected bilaterally in the DKO spinal cord. Consistently, microstimulation of 1 side of the motor cortex evoked electromyogram responses only in the contralateral forelimb muscles of the wild-type mice, whereas the same stimulation evoked bilateral responses in the DKO mice. The functional consequences of the CST defects in the *Sulf1/2* DKO mice were examined using the grid-walking, staircase, and single pellet-reaching tests, which have been used to evaluate motor function in mice. Compared with the wild-type mice, the *Sulf1/2* DKO mice showed impaired performance in these tests, indicating deficits in motor function. These findings suggest that disruption of *Sulf1/2* genes leads to both anatomical and functional defects of the CST.

## Introduction

The corticospinal tract (CST) plays a critical role in controlling voluntary movement (Lemon, 2008; Welniarz et al., 2017a). It is the longest tract in the central nervous system, originating in layer 5 pyramidal neurons in the sensorimotor cortex and terminating in the spinal cord. In rodents, the CST fibers pass through the internal capsule and cerebral peduncle, extend onto the ventral medulla, cross the midline at the pyramidal decussation dorsally, and further descend in the dorsal funiculus of the spinal cord contralaterally. Before reaching the spinal cord, they send collateral branches to the striatum, superior colliculus, red nucleus, pontine gray nucleus, and dorsal column nuclei (Wang et al., 2018). In the spinal cord, the CST fibers project to the dorsal and intermediate portions of the gray matter and innervate interneurons, which in turn control motor neurons (Lemon, 2008; Welniarz et al., 2017a).

Because the CST axons extend throughout the brain during development, a number of axon guidance molecules are required to navigate them to their targets correctly (Canty and Murphy, 2008; Leyva-Díaz and López-Bendito, 2013; Welniarz et al., 2017a). For example, Slit and its receptor Robo guide the axons by preventing them from entering the hypothalamic area and crossing the midline. Netrin-1 and its receptors DCC/Unc5, Sema6A and its receptors Plexin A3/A4, and the cell adhesion molecules L1 and NCAM are implicated in forming the pyramidal decussation (Dahme et al., 1997; Cohen et al., 1998; Fransen et al., 1998; Finger et al., 2002; Rolf et al., 2002; Faulkner et al., 2008; Rünker et al., 2008). In the spinal cord, Wnt and its receptor Ryk promote caudal growth (Liu et al., 2005), whereas ephrin and its receptor Eph are involved in topographical branching and innervation (Dottori et al., 1998; Kullander et al., 2001a, b; Yokoyama et al., 2001).

The heparan sulfate endosulfatases, Sulfatase 1 (Sulf1) and Sulfatase 2 (Sulf2), are extracellular enzymes that remove sulfate groups at the 6-*O* position of the glucosamine in heparan sulfate (HS) (Lamanna et al., 2007). Because Sulf-mediated desulfation occurs mainly in the highly sulfated regions of HS, which are required for interaction with signaling molecules, Sulfs can regulate cellular signaling positively or negatively. Previously, we showed that *Sulf1/2* double-knockout (DKO) mice have defects in the CST, whereas *Sulf1* or *Sulf2* single-knockout (KO) mice appear normal (Okada et al., 2017). Mechanistically, in *Sulf1/2* DKO mice, abnormal accumulation of Slit2 protein in the basement membrane of the ventral brain surface, which is caused by the increase in 6-*O*-sulfated HS, results in aberrant dorsal deflection of the CST axons on the lateral surface of the midbrain. Most of the axons that extend abnormally in the dorsal direction return to the medulla, whereas a part of them enter the superior and inferior colliculi. In the medulla, the CST fibers are defasciculated and positioned more laterally when compared with those of controls. Because DKO mice on a C57BL/6 background die within a day of birth, we could not assess the postnatal development of the CST. Specifically, it remained unclear whether pyramidal decussation and spinal cord projection occur normally.

Recently we found that for unknown reasons, *Sulf1/2* DKO mice survived into adulthood after outcrossing to the CD-1/ICR strain. Thus, we examined the CST trajectory in the adult *Sulf1/2* DKO brain using mice on a mixed genetic background. We found that the abnormal dorsal projection of the CST fibers on the midbrain surface that is observed in the embryonic brain is present in the adult brain. In addition, the DKO mice have abnormal pyramidal decussation and aberrant bilateral projections in the spinal cord. Consistently, stimulation of 1 side of the motor cortex evokes bilateral responses in the forelimb muscles of the DKO mice. Furthermore, we found that the DKO mice perform poorly in skilled reaching and grasping tasks, indicating that they have impaired motor movements.

## Materials and Methods

### Mice

*Sulf1* and *Sulf2* KO mice were generated using homologous recombination in 129/Ola-derived ES cells and maintained on a C57BL/6N background by mating offspring of mice that had been backcrossed to C57BL/6N for 5 successive generations (N5 generation), as described previously (Nagamine et al., 2012). These mice were mated with the outbred CD-1/ICR strain (F1 generation), and the offspring was further mated with the N5 generation of C57BL/6N (F2 generation). In this study, offspring of F1 or F2 on a mixed genetic background of C57BL/6N and CD-1/ICR (≒50%:50% or 75%:25%) were used. The genotypes were determined by PCR of genomic DNAs isolated from the tails. Wild-type and DKO mice (both from the mixed genetic background) were used, unless otherwise stated. All animal experiments were approved by and performed according to the guidelines of the Animal Care and Use Committee of the University of Tsukuba.

### Preparation of the Brain and Spinal Cord Sections

Mice were deeply anesthetized by intraperitoneal (i.p.) injection of an excess amount of pentobarbital and transcardially perfused with 4% paraformaldehyde (PFA) in phosphate-buffered saline (PBS). The brain and spinal cord were dissected and immersed in the same fixative overnight at 4°C. After being rinsed 3 times in PBS, the tissues were immersed in 20% sucrose in PBS for cryoprotection and embedded in Tissue-Tek O.C.T. compound (Sakura Finetek Japan, Tokyo, Japan). Coronal sections (50 μm thick) were cut on a cryostat (CM 1850; Leica Biosystems, Wetzlar, Germany), and the sections were collected in PBS. Free-floating sections were washed three times in PBS and once in PBS with 0.1% Tween-20 (PBT) and serially dehydrated through 25–80% methanol/PBT and then 80% methanol/20% dimethyl sulfoxide (DMSO). To block endogenous peroxidase activity, the sections were incubated in 3% H_2_O_2_/80% methanol/20% DMSO for 30 min. After rehydration, the sections were subjected to further processing.

### Immunohistochemistry

Protein kinase C gamma (PKCγ) immunohistochemistry was done as previously described (Okada et al., 2019). Briefly, the brain sections of mice (16–56 weeks old) were incubated twice overnight with anti-PKCγ antibody (1:200; Frontier Institute, Hokkaido, Japan) in 0.5% blocking reagent (Roche Diagnostics, Mannheim, Germany) in PBT at 4°C. The sections were washed with PBT for 15 min six times and then incubated with biotin-conjugated anti-rabbit IgG antibody (1:600; Vector Laboratories, Burlingame, CA, United States) in 0.5% blocking reagent in PBT for 2 h. After being washed with PBT for 15 min five times and with Tris-buffered saline (TBS) supplemented with 0.1% Tween-20 (TBST) for 15 min, the sections were incubated with avidin-biotin peroxidase complex (ABC; Vectastain Elite ABC kit; Vector Laboratories) for 30 min. The sections were washed with 1% Tween-20/TBS for 20 min, and then with TBST for 20 min twice, and were thereafter kept in the same solution at 4°C overnight. The sections were incubated with 3,3′-diaminobenzidine (DAB; Vector Laboratories) for 10 min. All steps were performed at room temperature unless otherwise indicated.

### Anterograde Tracing of the CST

Anterograde tracing of the CST was done using biotinylated dextran amine (BDA) as follows. Adult mice of either sex (9–45 weeks old, 17.1–53.3 g) were anesthetized by i.p. injection of pentobarbital (50 mg/kg body weight), and placed in a stereotaxic frame (David Kopf Instruments, Tujunga, CA, United States). After the scalp was incised, a burr hole was made using a dental drill. After the dura was removed, a Neuros syringe (model 75RN; Hamilton Laboratories, Reno, NV, United States) was inserted into the target area in the right sensorimotor cortex. The stereotaxic coordinates (anterior-posterior [AP] to the bregma; medial-lateral [ML] from the midline; dorsal-ventral [DV] from the pial surface, in mm) were +1.2 AP, +1.5 ML, 0.7 DV for the forelimb area, and −1.2 AP, +1.0 ML, 0.7 DV for the hindlimb area. Systematic injection to the sites at +1.2, 0, and −1.2 AP and from 0.5 to 3.0 ML was also performed. BDA (10,000 MW, lysine-fixable; Molecular Probes, Eugene, OR, United States) was dissolved in PBS (10%) and 0.5 μl was injected over 5 min. Five min after the injection was completed, the tip was slowly removed from the cortex, and the mice were returned to their cages.

To examine the labeled fibers in the brain and spinal cord, the mice were transcardially perfused with 4% PFA/PBS at 8 and 15 days after the injection, respectively. The brain sections, prepared as described previously, were incubated with ABC for 30 min and subjected to the DAB reaction for 20 min at room temperature. The spinal cord sections (approximately C2 and L1–3 levels) were further subjected to tyramide signal amplification (TSA). For this, after ABC treatment, the sections were incubated with biotin tyramide (TSA Biotin system; PerkinElmer, Waltham, MA, United States) for 10 min. After washing with TBST, the sections were again incubated with ABC for 30 min. After washing with TBST, the signals were visualized by incubation with DAB for 20 min at room temperature. The sections were mounted on MAS-coated slide glasses (Matsunami Glass Industry, Osaka, Japan), dehydrated through an ethanol series, and cleared in xylene, and the coverslips were mounted using Poly-mount (Polysciences, Warrington, PA, United States). Images were observed and recorded using microscopes (Axioplan 2; Carl Zeiss Microscopy, Jena, Germany and BZ-8000; Keyence, Osaka, Japan). In all cases, the precise location of the BDA injection site in the target area was confirmed by examining the stained sections.

### 3D Reconstruction

3D reconstruction of the serial sections was done as described previously (Okada et al., 2019). Briefly, the 2D images of the serial sections were aligned using AutoAligner alignment software (Bitplane, Zürich, Switzerland) on the basis of the shape of the sections and the location of the signals. Stacks of the aligned images were imported into Imaris software (Bitplane) and transformed into 3D images.

### Quantification of Labeled Fibers

To examine the distribution of the BDA-labeled fibers in the spinal cord, the BDA signals in the spinal cord sections were quantified using ImageJ software^1^. Digital images were obtained using a microscope (Axioplan 2) with a 5 × objective lens and a cooled CCD camera (VB-7010, Keyence). Bright-field images were converted to 8-bit grayscale, and the colors of the images were subsequently inverted. The threshold of the background signal was determined using the triangle algorithm (Zack et al., 1977) in the Auto Threshold method of ImageJ software. The integrated density, the sum of the values of the signals above the threshold, was measured for each region of interest (ROI). To quantify the signals in the descending tract, ROIs were drawn to outline the contralateral dorsal funiculus (cDF) and ipsilateral lateral funiculus (iLF) (Figure 6A) using dark-field illumination, which can highlight the border between the white and gray matter. Percent integrated density was calculated as the ratio of the integrated density in each ROI to the sum of the integrated density in the cDF and iLF of the cervical and lumbar cord regions. To quantify the signal in the contralateral and ipsilateral side, the gray matter was divided into right and left halves. To quantify the signal along the dorsoventral axis, the gray matter was divided into quarters and ROIs were drawn to outline the dorsal quarter, intermediate half, and ventral quarter on both sides (Figure 6A). Percent integrated density was calculated as the ratio of the integrated density in each ROI to the sum of the integrated density in the gray matter.

### Intracortical Microstimulation and Electromyography

Intracortical microstimulation and electromyogram (EMG) recordings were performed using the methods reported previously (Li and Waters, 1991; Serradj et al., 2014; Gu et al., 2017; Ueno et al., 2018) with some modifications. Briefly, after injection of atropine sulfate (0.05 mg/kg body weight, i.p.), the mice were anesthetized by injection of ketamine (100 mg/kg body weight, i.p.). During surgery, isoflurane inhalation was used in conjunction. One-fifth the amount of ketamine was further added when a pain-induced reflex or spontaneous movement was observed during surgery and recording. The mouse was placed in a stereotaxic frame (Narishige, Tokyo, Japan) and craniotomy was performed. A polyurethane-coated coaxial microelectrode (200 μm diameter, 50 kΩ tip resistance; Unique Medical, Tokyo, Japan) was inserted into the motor cortex (+0.75 AP, +1.5 ML, 0.6–1.0 DV) and 14 square-pulses of current stimulations (200-μs duration, 3-ms intervals, 20–100 μA) were delivered every 1 s using a pulse generator (SEN-7103M; Nihon Kohden, Tokyo, Japan) and an isolator (SS-401J, Nihon Kohden). When forelimb movements were observed, the EMG responses were recorded differentially using nichrome wire electrodes (tip 0.5 mm deinsulated) inserted into the bilateral biceps and triceps muscles with an amplifier (MEG-6108, Nihon Kohden) with low- and high-frequency cutoffs of <150 Hz and >3 kHz, respectively. EMG responses to 4 square-pulses of current stimulation (200-μs duration, 3-ms intervals, every 550 ms, 20–100 μA) to the motor cortex were recorded using an AD converter (model 1401 plus; Cambridge Electronic Design, Cambridge, United Kingdom) and Spike2 software (ver. 7.2; Cambridge Electronic Design). The EMGs were rectified and averaged 500 times with triggering by microstimulations. At the end of the experiment, electric lesions (20 μA, 10 s) were made to mark the location of the recording sites. The mouse was transcardially perfused with 4% PFA under deep anesthesia and the brain was histologically examined.

### Grid-Walking Test

The grid-walking test was performed essentially as described previously (Starkey et al., 2005). Male mice (11–12 weeks old) were placed on an elevated wire grid (32 × 20 cm square with 11 × 11 mm grids, placed 50 cm from the floor) and allowed to explore freely for 3 min. Behavior was recorded using a digital camera (EX-FR100; Casio, Tokyo, Japan) at 30 frames per sec and scored later. An investigator blinded to the mouse genotype counted the number of foot-fault errors, which were scored when one of the limbs fell below the grid surface (Figure 8A). Because the mice moved their forelimbs four times more frequently than their hindlimbs in this test, we independently analyzed the first 200 steps taken by the forelimbs and the first 50 steps taken by the hindlimbs on both sides. The foot-fault rate (% foot fault) was calculated by dividing the numbers of the foot-fault errors by 200 for the forelimbs and 50 for the hindlimbs.

### Staircase Test

The staircase test was performed as described previously with minor modifications (Montoya et al., 1991; Baird et al., 2001). A staircase apparatus for mice (model 80301) was purchased from Melquest (Toyama, Japan). It consists of a start chamber with a clear, hinged lid and a narrow corridor with a central platform and a double staircase (Figure 8C′). The central platform extends along the length of the corridor, and a removable double staircase with 8 steps on each side can be inserted into the space between the platform and walls. Food pellets (Dustless Precision Pellet, 20 mg; Bioserv, Flemington, NJ, United States) are baited in the shallow well of each step (Figure 8C), and the mouse retrieves the pellets on either side only with the forelimb of the same side because it cannot turn around in the corridor.

At the beginning, male mice (11–12 weeks old) were familiarized to the food pellets by 55 mg per g body weight per d being placed in their cages on 3 consecutive days. On the next day, the mice were habituated to the staircase box for 30 min with four pellets baited along the platform as well as two pellets baited on each step of both sides. From this day, the mice were deprived of food for 18–20 h before the daily test session. Over the next 4 days (test session), the mice were placed in the apparatus with single pellets baited on each well in the lower seven steps on both sides. During the test time (30 min), the mice were allowed to enter the corridor and reach, retrieve, and eat the pellets freely. At the end of each session, an experimenter checked the number and place of the remaining pellets to calculate the following values and to evaluate skilled function. Pellets baited on the second step were not scored because many of the mice used their tongues to retrieve pellets (wild-type, *n* = 5/6; DKO, *n* = 6/6), but pellets were still presented on these steps because otherwise the mice seemed to lose their motivation to reach the pellets in the lower steps. The “number of pellets collected” (scores from 0 to 12) was calculated by subtracting the number of the remaining pellets from the total number of pellets baited from the third to eighth well on both sides, which indicated the number of pellets successfully eaten by the mouse during the session. The “maximum distance reached” (scores from 0 to 6) indicated the deepest well reached by the mouse (the larger of the right or left) regardless of whether it ate, dropped, or knocked down the pellets; scores 1–6 indicate that the mouse reached the third to eighth well, respectively. The “success rate” was calculated by dividing the “number of pellets collected” by the sum of the wells reached by the mouse on both sides. The mouse’s behavior was recorded using a digital camera for later analysis.

### Single Pellet-Reaching Test

The single pellet-reaching test was performed as described previously, with minor modifications (Farr and Whishaw, 2002; Chen et al., 2014). The apparatus was a clear box made of plexiglass (20 cm high, 15 cm deep, 8.5 cm wide, measured from the outside, and 5 mm thick) that had three vertical slits (13 cm high, 0.5 cm wide): one central slit on one side and two lateral slits (2.5 cm lateral to the center) on the opposite side. At the beginning, male mice (13–15 weeks old) were habituated to the food pellets (Dustless Precision pellet, 20 mg; Bioserv) for 2 days, by 55 mg per g body weight per day being placed in their cages. On the next day, the mice were familiarized to the apparatus with 20 pellets placed in it for 20 min. From this day, the amount of laboratory chow was adjusted to maintain approximately 90% of the free-feeding weight. A shaping session started on the following day. The mice were placed in the apparatus with a tilted food tray affixed to the front of the center slit and filled with pellets. In this session, the mice used both forelimbs to reach for the pellets. The shaping session was finished when the mouse performed 20 reaching attempts within 20 min. If the mouse did not perform 20 reaching attempts within 20 min, the shaping session was conducted again on the next day. If the mouse could not complete shaping within 5 days, it was excluded from further testing (1 of 10 wild-type mice and 1 of 11 DKO mice). The preferred limb was determined by counting which forelimb the mice used more frequently (>50%) in the shaping session. The test sessions started the day after shaping and lasted for 8 days, one session per day. A wild-type mouse died for unknown reasons after completing the shaping session and thus was excluded from further analysis.

In the test sessions, the mice were placed in the apparatus with the double-slit side facing downward. A holding plate (10 mm high) was affixed to the front wall of the apparatus. To place a pellet at the same position consistently, two divots were made: 6.5 mm from the front wall and 4 mm medial to the center of each lateral slit. A single pellet was baited on the divot of the preferred side of each mouse. Because the mice pronated the forelimb medially to reach for the pellet, the medial displacement of the divot encouraged them to reach with the preferred limb. A daily test session was finished when 30 reaching attempts were performed or the time limit of 20 min was exceeded. Behavior was recorded using a digital camera at 30 frames per sec for later analysis. Reaching attempts were classified into four categories: success, drop, loss, and failure. “Success” means that the mouse successfully grasped the pellet and brought it into its mouth. “Drop” means that the mouse grasped the pellet and dropped it inside the chamber before putting it into its mouth. “Loss” means that the mouse grasped the pellet and dropped it outside the chamber and thus could not eat it. “Failure” means that the mouse missed, touched, or knocked the pellet and failed to grasp it eventually. “Failure” also includes drawing the pellet without grasping regardless of whether the mouse ultimately brought the pellet into its mouth. The rates for success, drop, loss, and failure were calculated by dividing the numbers of success, drop, loss, and failure by the respective total attempts.

To examine the reaching movement trajectories of the forelimbs, five successful reaches of each mouse were analyzed using the recorded video. All the mice except 1 *Sulf1/2* DKO mouse achieved at least five successful reaches during the total test period. If a mouse achieved five or more successful reaches on the last test day, the first five successful reaches on the day were analyzed. If a mouse achieved less than 5 successful reaches on the last test day, successful reaches on the previous days were included for analysis. The positions of the distal tip of the second digit, second metacarpophalangeal joint, and wrist were marked separately in single frames and each trajectory was calculated using MTrackJ^2^ (Meijering et al., 2012), a plugin for ImageJ software. To analyze the velocity profiles of the reaching movements, the positions of the distal tip of the second digit in the five consecutive frames (P_1_–P_5_) in a successful reach, in which P_3_ corresponds to the points of pellet grasping, were marked in the video, and the velocity between 2 points (*v*_i_ means the velocity between P_i_ and P_i+__1_) was measured.

### Rotarod Test

The rotarod test was performed using a rotarod apparatus (ENV-577M; Med Associate Inc, Fairfax, VT, United States). Five mice were put on a rod and the length of time that each remained on the rotating rod was measured. The mice were subjected to a total of 6 sessions on consecutive days (three sessions per day), with accelerating speed (4–40 rpm) over 5 min.

### Open Field Test

The open field test was performed using a square arena [OF-36(M)SQ; 500 × 500 mm, wall height of 400 mm; Muromachi Kikai, Tokyo, Japan] and a video tracking system (ANY-maze; Stoelting, Wood Dale, IL, United States). Each mouse was allowed to walk in the arena freely for 30 min, and the total distance traveled was measured.

### Hot Plate Test

The hot plate test was performed as previously described, with minor modifications (O’Callaghan and Holtzman, 1975). A male mouse (17–18 weeks old) was placed on a hot plate maintained at 55°C (FHP-450; Tokyo Garasu Kikai, Tokyo, Japan), and the latency to jump, paw-shaking or paw-licking was recorded.

### Statistical Analysis

All the statistical tests were performed using Prism 4.0c (GraphPad Software; San Diego, CA, United States). Statistical significance in the staircase test, single pellet-reaching task, and BDA tracing study was evaluated using one-way or two-way repeated-measures analysis of variance (ANOVA) or two-way ANOVA with Bonferroni *post hoc* tests. Statistical significance in the grid walking test, hot plate test, and velocity analysis was evaluated using the Mann–Whitney *U* test.

## Results

### CST Defects in the Adult *Sulf1/2* DKO Brain Revealed by PKCγ Staining

**FIGURE 1:**
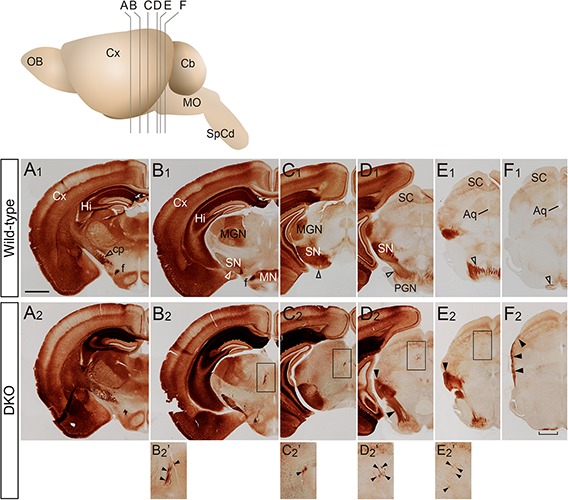
PKCγ staining images of the adult brain. **(A–F)** Coronal sections of wild-type **(A_1_–F_1_)** and *Sulf1/2* DKO **(A_2_–F_2_)** brains through the cerebral peduncle (cp) to the pons are shown. The positions of sections **(A–F)** in the brain are shown in the upper panel. The open and filled arrowheads indicate the normal and abnormal projections of CST fibers, respectively. In the *Sulf1/2* DKO brain, a small number of fibers projected abnormally through the thalamus to the midbrain (**B_2_′–E_2_′**, showing magnified images in the boxed regions in **B_2_–E_2_**, respectively). In the *Sulf1/2* DKO brain, misdirected fibers were found on the surface of the midbrain **(E_2_–F_2_)** and the pyramidal tract was thinner and broader (**F_2_**, bracket) than that in the wild-type control. Aq, aqueduct; Cb, cerebellum; Cx, cerebral cortex; f, fornix; Hi, hippocampus; MGN, medial geniculate nucleus; MN, mammillary nucleus; MO, medulla oblongata; OB, olfactory bulb; PGN, pontine gray nucleus; SC, superior colliculus; SN, substantia nigra; SpCd, spinal cord. The scale bars indicate 1.0 mm **(A_1_–F_1_,A_2_–F_2_)** and 500 μm **(B_2_′–E_2_′)**.

**FIGURE 2:**
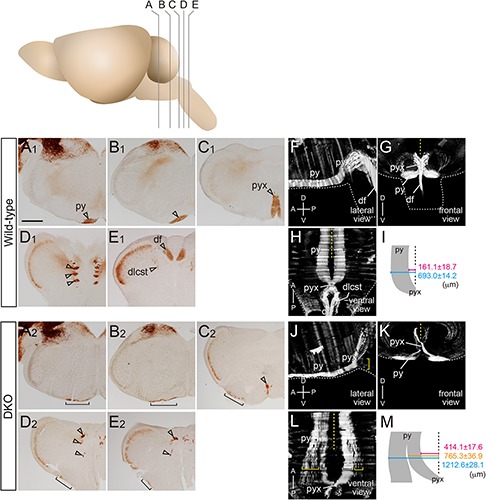
PKCγ staining images and their 3D reconstruction. **(A–E)** Coronal sections of wild-type **(A_1_–E_1_)** and *Sulf1/2* DKO **(A_2_–E_2_)** brains from the medulla to the anterior spinal cord are shown. The positions of coronal sections **(A–E)** in the brain are shown in the upper panel. The open arrowheads in **(A–E)** indicate the normal projections of CST fibers. **(F–H,J–L)** 3D reconstruction of PKCγ-positive fibers. Lateral **(F,J)**, frontal **(G,K)**, and ventral **(H,L)** views of the 3D images are shown. The CST fibers formed a pyramidal tract (py) on the ventral surface of the medulla. At the pyramidal decussation (pyx), most fibers entered the contralateral dorsal funiculus (df), whereas a small portion descended laterally to the dorsal funiculus as the dorsolateral CST (dlcst; **A_1_–E_1_,F–H**). In the *Sulf1/2* DKO brain, the pyramidal tract became thinner and broader (**A_2_–D_2_**, brackets). The pyramidal tract gradually split into medial and lateral bundles **(K,L)**. The medial bundle (white brackets, **K,L**) entered the contralateral dorsal funiculus, whereas the lateral bundle (yellow brackets, **J–L**) extended on the ventrolateral surface of the ipsilateral spinal cord. The yellow and white dotted lines in **(F–H,J–L)** indicate the midline and contour of the brain, respectively. Anterior-posterior (A-P) and dorsal-ventral (D-V) body axes are shown. **(I,M)** Illustrate the ventral views of the CST fibers and their distance from the midline (dotted lines). The scale bars indicate 600 μm **(A–E)** and 1.0 mm **(F–H,J–L)**.

To examine the CST trajectory in the adult brain, we first performed immunohistochemistry for PKCγ, a marker for CST fibers in the adult mouse brain (Mori et al., 1990; Ding et al., 2005; Joshi et al., 2008) because this staining is useful to overview all the CST fibers. In the wild-type mice, the CST fibers formed the cerebral peduncle in the caudal forebrain (Figure 1A_1_), passed ventrally (Figures 1B_1_,C_1_), and extended medially toward the pons (Figures 1D_1_–F_1_). In the medulla, the CST fibers formed the pyramidal tract (Figure 2A_1_), extended toward the midline (Figure 2B_1_), decussated (Figures 2C_1_,D_1_), and entered the contralateral dorsal funiculus of the spinal cord (Figure 2E_1_). In the DKO mice, the CST fibers appeared almost normal up to the ventral midbrain (Figures 1A_2_–C_2_), whereas at the level of the pons, the abnormal fibers extended dorsally on the surface of the midbrain (Figures 1D_2_–F_2_). In addition, a small number of the fibers projected toward the superior colliculus through the thalamus (Figures 1B_2_–E_2_, 1B_2_′–E_2_′, arrowheads). In the medulla, the pyramidal tract became flattened and laterally widened (Figures 2A_2_,B_2_). A part of the fibers near the midline crossed the midline, whereas others located more laterally extended to the ventrolateral surface of the medulla (Figures 2C_2_–E_2_).

To examine the midline crossing, we reconstructed 3D images of the CST trajectory from the PKCγ-stained brain sections. In the wild-type mice, the CST fibers ran close to the midline and crossed contralaterally at the pyramidal decussation (Figures 2F–H). Most of the fibers entered the dorsal funiculus, whereas a few fibers, which form the dorsolateral CST (Steward et al., 2004), descended in a more lateral position (Figures 2E_1_,H, dlcst). In contrast, the *Sulf1/2* DKO mice showed defects in the pyramidal decussation (Figures 2J–L and Supplementary Figure S1). First, the CST fibers were located more laterally than those in the wild-type mice: the largest distances between the midline and the medial border of the CST fibers in the medulla were 161.1 ± 18.7 μm in the wild-type mice (*n* = 6 CSTs from three mice; Figure 2I) and 414.1 ± 17.6 μm in the DKO mice (n = 6 CSTs from three mice; Figure 2M), whereas those between the midline and the lateral borders of the CST fibers were 693.0 ± 14.2 μm in the wild-type mice and 1212.6 ± 28.1 μm in the DKO mice (Figures 2I,M). Secondly, in the DKO mice, the laterally deviated fibers descended ipsilaterally without midline crossing, whereas the medially located fibers crossed the midline (Figures 2K,L). The outermost fibers that crossed the midline in the DKO mice were 765.3 ± 36.9 μm apart from the midline (Figure 2M), which was close to the distance of the outermost fibers from the midline in the wild-type mice.

### BDA Tracing of the CST Fibers in the *Sulf1/2* DKO Brain

**FIGURE 3:**
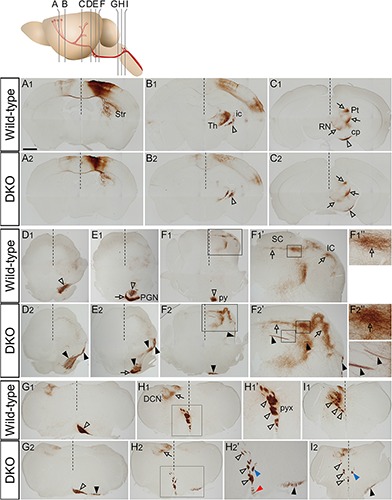
BDA tracing of CST fibers. **(A–I)** Coronal sections of BDA-injected wild-type **(A_1_–I_1_)** and *Sulf1/2* DKO **(A_2_–I_2_)** brains are shown. The positions of sections **(A–I)** in the brain are shown in the upper panel. The open and filled arrowheads indicate the normal and abnormal projections of CST fibers, respectively. The open arrows indicate collateral branches. The dotted lines indicate the midline. **(F${}_{1}^{\prime }$,F${}_{2}^{\prime }$,F${}_{1}^{\prime \prime }$,F${}_{2}^{\prime \prime }$,H${}_{1}^{\prime }$,H${}_{2}^{\prime }$)** show magnified images in the boxed regions in **(F_1_,F_2_,F${}_{1}^{\prime }$,F${}_{2}^{\prime }$,H_1_,H_2_)**, respectively. **(A_1_,A_2_)** Illustrate BDA injection sites in the motor cortex. The labeled CST fibers passed through the internal capsule (ic) and cerebral peduncle (cp) with collateral branches projecting to the pretectum (Pt) and red nucleus (RN) in both the wild-type and the *Sulf1/2* DKO brains **(B_1_,B_2_,C_1_,C_2_)**. In the *Sulf1/2* DKO brain, the aberrant fibers were found on the surface of the midbrain **(D_2_–F_2_)**. In the DKO medulla, some fibers reached the pyramidal decussation (pyx), whereas others deviated laterally and descended ipsilaterally in the ventrolateral position (**G_2_–I_2_**, black arrowheads). At the pyramidal decussation, some fibers crossed the midline normally and entered the contralateral dorsal funiculus (**G_2_**,**H${}_{2}^{\prime }$**,**I_2_**; open arrowheads). A small portion of fibers turned dorsally but did not cross the midline and entered the ipsilateral dorsal funiculus (**H${}_{2}^{\prime }$**,**I_2_**; blue arrowheads). Other aberrant fibers descended in the ipsilateral ventral funiculus (**H${}_{2}^{\prime }$**; red arrowhead). DCN, dorsal column nuclei; IC, inferior colliculus; PGN, pontine gray nucleus; py, pyramidal tract; SC, superior colliculus; Str, striatum; Th, thalamus. The scale bars indicate 1.0 mm **(A_1_–C_1_,A_2_–C_2_)**, 850 μm **(D_1_–F_1_,D_2_–F_2_)**, 300 μm **(F${}_{1}^{\prime }$,H${}_{1}^{\prime }$,F${}_{2}^{\prime }$,H${}_{2}^{\prime }$)**, 500 μm **(G_1_–I_1_,G_2_–I_2_)**, and 100 μm **(F${}_{1}^{\prime \prime }$,F${}_{2}^{\prime \prime }$)**.

Given that PKCγ staining is also positive in non-CST neurons, to see the CST trajectory more specifically, we next performed anterograde tracing of the CST fibers using BDA in wild-type (*n* = 15) and *Sulf1/2* DKO (*n* = 21) mice. BDA was stereotaxically injected into the primary motor area and subsequently the CST trajectories were visualized by detecting the BDA-positive fibers by means of the avidin-biotin peroxidase complex (ABC) and DAB reaction. In both the wild-type and the DKO brains, the labeled fibers extended into the internal capsule and cerebral peduncle, with their branches projecting to the striatum, thalamus, red nucleus, and pretectum (Figures 3A_1_–C_1_,A_2_–C_2_). At the level of the pons, in the wild-type mice, the CST fibers turned medially from the lateral surface of the brain (Figure 3D_1_), ran beneath the pons, sent branches to the pons (Figure 3E_1_, open arrow), and descended caudally to form the pyramidal tract (Figure 3F_1_). In the DKO mice, abnormal CST fibers were found on the lateral surface of the midbrain (Figures 3D_2_–F_2_). In the caudal pons, a flattened and widened pyramidal tract was observed (Figure 3F_2_).

In the tectum of the wild-type mice, BDA-positive collaterals were observed in the superior colliculus (Figure 3F_1_). These fibers ran through the thalamus and formed a dense clump at the lateral aspect of the superior colliculus (Figures 3F_1_′,F_1_″) and subsequently projected medially into the intermediate layer of the superior colliculus (Figure 3F_1_′). In the DKO brain, a similar distribution of the labeled fibers in the intermediate layer was observed, although the density of the fibers was much higher than in the wild-type mice (Figures 3F_2_,F_2_′,F_2_″). In addition, a thick bundle of the labeled fibers traversed the deep layer of the superior colliculus (*n* = 12/21, Figures 3F_2_′,F_2_″).

In the medulla of the wild-type mice, the labeled fibers crossed the midline (Figures 3H_1_,H_1_′,I_1_). In the DKO mice, the labeled fibers split into 2 bundles. The lateral bundle extended in the ventrolateral position ipsilaterally (*n* = 21/21; Figures 3G_2_–I_2_, black arrowheads), whereas the medial bundle reached the midline at the pyramidal decussation and most of the fibers crossed the midline (Figures 3H_2_–I_2_, open arrowheads). In some cases, a few fibers reached the midline and returned to the ipsilateral side (*n* = 8/21; Figures 3H_2_–I_2_, blue arrowheads); projection to the ipsilateral dorsal funiculus was also observed in the wild-type mice (*n* = 2/15, data not shown), but the frequency and number of these fibers were higher in the DKO mice. Other fibers entered the ventral funiculus on the ipsilateral side in the DKO mice (*n* = 9/21; Figure 3H_2_′, red arrowhead); a few fibers were also seen in the ipsilateral ventral funiculus of the wild-type mice (*n* = 2/15, data not shown). These analyses clearly demonstrated the presence of midline crossing errors in the *Sulf1/2* DKO mouse (see Supplementary Table S1 for summary).

**FIGURE 4:**
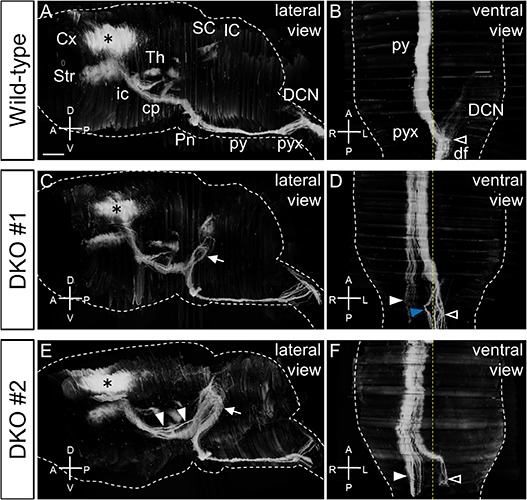
3D images of the BDA-labeled CST. **(A–F)** Representative images from the wild-type **(A,B)** and 2 *Sulf1/2* DKO **(C–F)** mice are shown. DKO mouse #1 **(C,D)** is the same as the one shown in Figure 3. Lateralviews of the whole brain **(A,C,E)** and ventral views of the medulla **(B,D,F)** are shown. The yellow and white dotted lines indicate the midline and contour of the brain, respectively. The asterisks indicate BDA injection sites. In the wild-type brain **(A,B)**, a whole image of the CST from the motor cortex (Cx) to the contralateral dorsal funiculus (df; open arrowhead) was successfully obtained. DKO mice showed abnormal looping of the labeled fibers on the surface of the midbrain (**C,E**; arrows). In the cerebral peduncle (cp) of *Sulf1/2* DKO mouse #2, the CST fibers were slightly defasciculated (**E**, filled arrowheads). In the medulla of the DKO mice, laterally located fibers projected ipsilaterally to the spinal cord (**D,F**; filled arrowheads). At the pyramidal decussation (pyx), almost all the fibers that extended to the midline crossed the midline in *Sulf1/2* DKO mouse #2 (**F**, open arrowhead), whereas a part of the fibers approached the midline but entered the ipsilateral dorsal funiculus in *Sulf1/2* DKO mouse #1 (**D**, blue arrowhead). Anterior-posterior (A-P), dorsal-ventral (D-V), and right-left (R-L) body axes are shown. DCN, dorsal column nuclei; ic, internal capsule; IC, inferior colliculus; Pn, pons; py, pyramidal tract; SC, superior colliculus; Str, striatum; Th, thalamus. The scale bars indicate 1.0 mm **(A,C,E)** and 600 μm **(B,D,F)**.

Next, we performed 3D reconstruction of the BDA-labeled CST fibers in the whole brain. In the wild-type mice, the CST fibers descended through the internal capsule, cerebral peduncle, and pyramidal tract and crossed the midline at the pyramidal decussation (Figures 4A,B). It was apparent that the CST fibers entered the contralateral dorsal funiculus (Figure 4B). In addition to the main tract, collateral branches and their fiber terminals were observed in the striatum, thalamus, superior colliculus, pons, and dorsal column nuclei (Figure 4A; Catsman-Berrevoets and Kuypers, 1981; O’Leary et al., 1990; Lévesque et al., 1996; Wang et al., 2018). In the DKO mice, the most prominent abnormality was the U-shaped trajectory in the midbrain (*n* = 21/21; Figures 4C,E; arrows). After the fibers extended dorsally toward the superior colliculus, most of them returned to the pons. These data clearly showed that the aberrant U-shaped detour of the CST fibers observed in the embryonic DKO brain persisted into adulthood in all the DKO mice. In the 3D reconstruction images, variable decussation defects were observed in the DKO mice. Consistent with the PKCγ staining results, the lateral bundle extended ipsilaterally (Figures 4D,F, white arrowheads), whereas the medial bundle reached the midline and some of the fibers crossed the midline (Figures 4D,F, open arrowheads) and others returned to the ipsilateral side (Figure 4D, blue arrowhead). Furthermore, defasciculation of the CST fibers in the cerebral peduncle was also observed (*n* = 9/21; Figure 4E, arrowheads). Information about all the mice analyzed and their phenotypes is summarized in Supplementary Table S1.

### Bilateral CST Fiber Projections in the Spinal Cord of *Sulf1/2* DKO Mice

**FIGURE 5:**
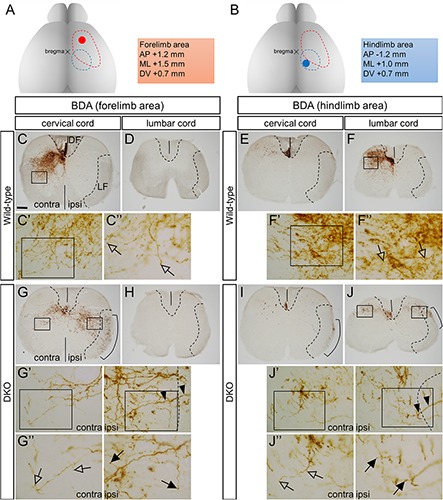
Projection of BDA-labeled CST fibers in the spinal cord. **(A,B)** BDA injection sites for the forelimb **(A)** and hindlimb **(B)** areas. The red and blue dotted lines encompass the forelimb and hindlimb areas, respectively. Stereotaxic coordinates for representative injection sites (red and blue spots) are shown on the right. **(C–J)** Images of the transverse sections of the cervical and lumbar spinal cord regions of the wild-type **(C–F)** and *Sulf1/2* DKO mice **(G–J)** are shown. The dashed lines delineate the borders of the dorsal funiculus (DF) and ipsilateral lateral funiculus (LF). The solid lines indicate the midline. **(C′,C″,F′,F″,G′,G″,J′,J″)** Show the magnified pictures of the boxed area in **(C,C′,F,F′,G,G′,J,J′)**, respectively. In the wild-type mouse, the labeled CST fibers that descended in the contralateral DF projected to the dorsal horn and intermediate zone **(C,F)** and formed terminal arbors with bouton-like structures (**C″,F″**; open arrows). In the *Sulf1/2* DKO mouse, the labeled CST fibers descended in both the contralateral DF and the ipsilateral LF **(G,I,J)** and projected bilaterally in the gray matter **(G,J)**. The terminal arbors in both the contralateral (open arrows) and the ipsilateral gray matter (filled arrows) showed bouton-like structures **(G″,J″)**. The arrowheads in **(G′)** and **(J′)** show the fibers that entered the gray matter from the ipsilateral LF. The scale bar indicates 200 μm **(C–J)**, 35 μm **(C′,F′,G′,J′)**, and 20 μm **(C″,F″,G″,J″)**.

Next, we examined the projection of the CST fibers in the spinal cord. When BDA was injected stereotaxically into the forelimb area of the Primary motor cortex (M1) of the wild-type mice (Figure 5A; a representative stereotaxic coordinate, +1.2 AP, +1.5 ML, 0.7 DV, in mm), the labeled fibers were observed in the ventromedial portion of the dorsal funiculus in the cervical spinal cord on the contralateral side of the injection (Figure 5C). The fibers entered the intermediate portion of the gray matter (Figure 5C) and their terminals showed bouton-like structures (Figures 5C′,C″, open arrows). Thus, the CST fiber projection was almost exclusively confined to the contralateral side in the wild-type mice. Few signals reached the lumbar spinal cord (Figure 5D).

In the DKO mice, the labeled fibers descended in the lateral funiculus on the ipsilateral side in addition to in the dorsal funiculus on the contralateral side (Figure 5G). The fibers in the lateral funiculus entered the ipsilateral gray matter in a medial direction (Figure 5G′, arrowheads), whereas the fibers in the dorsal funiculus entered the contralateral gray matter in a ventrolateral direction (Figure 5G). In addition, a few fibers crossed the midline in the spinal cord (Supplementary Figure S2). The terminals of the fibers from both the lateral and the dorsal funiculi had bouton-like structures (Figure 5G″, arrows). Thus, the CST fiber projection was bilateral in the DKO mice. Few signals reached the lumbar spinal cord (Figure 5H), indicating that the CST fibers originating from the forelimb area projected to their inherent projection levels in the DKO mice.

When BDA was injected into the hindlimb area of the M1 of the wild-type mice (Figure 5B; a representative stereotaxic coordinate, −1.2 AP, +1.0 ML, 0.7 DV), the labeled fibers passed into the contralateral dorsal funiculus at the cervical level with few projecting into the gray matter (Figure 5E). At the lumbar level, the fibers in the dorsal funiculus projected into the dorsal portion of the gray matter (Figures 5F,F′,F″). In the DKO mice, the labeled fibers descended in both the ipsilateral lateral funiculus and the contralateral dorsal funiculus (Figures 5I,J). Projection into the gray matter was scarce in the cervical spinal cord (Figure 5I), whereas bilateral projection to the dorsal portion was observed in the lumbar spinal cord (Figures 5J,J′,J″). These findings suggest that the CST fibers of the DKO mice terminate at the appropriate levels of the spinal cord even though the trajectories are aberrant.

**FIGURE 6:**
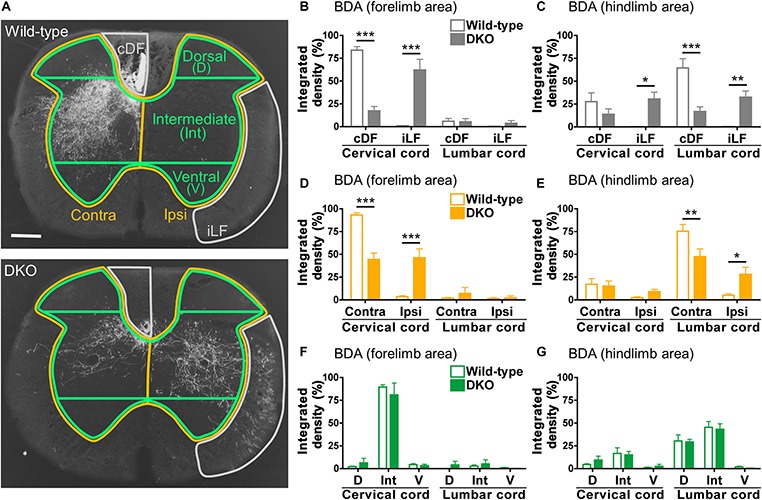
Quantitation of the BDA-labeled CST fibers in the spinal cord. **(A)** Regions of interest (ROIs) for quantifying the BDA-labeled fibers. The ROIs for the contralateral dorsal funiculus (cDF) and ipsilateral lateral funiculus (iLF) (outlined by gray lines); contralateral (Contra) and ipsilateral (Ipsi) gray matter (outlined by orange lines); and dorsal (D), intermediate (Int), and ventral (V) gray matter (outlined by green lines) are shown. Representative images of the cervical spinal cord from wild-type and *Sulf1/2* DKO mice that received BDA injection in the forelimb area (the same as the ones shown in Figures 5C,G) are shown. The scale bar indicates 200 μm. **(B–G)** The integrated density, the sum of the values of the signals above the threshold, was measured for each ROI, and its rate to the sum of the integrated density in all the ROIs analyzed in the cervical and lumbar spinal cord regions is shown as a percentage. The percent integrated density of the BDA signals after injection into the forelimb **(B,D,F)** and hindlimb **(C,E,G)** areas in the wild-type (*n* = 6) and DKO (*n* = 6) mice is plotted. In **(F,G)**, the integrated densities on both sides are combined and analyzed. Data shown are means ± SEMs. Statistical significance was calculated using two-way ANOVA with a Bonferroni *post hoc* test (^∗^*P* < 0.05, ^∗∗^*P* < 0.01, ^∗∗∗^*P* < 0.001).

To compare the differences in the CST projection between the wild-type and DKO mice precisely, we quantitated the distribution of the BDA-labeled fibers in the spinal cord. Because the extent of labeling differed among individuals, we measured the total number of signals present in the cervical or lumbar spinal cord and calculated the percentages of the signals in the regions of interest (ROIs). To this end, we used the integrated density, the sum of the values of the signals above the threshold, which was determined using ImageJ software. The ratio of the labeled fibers in each ROI among all the fibers in the white or gray matter was shown as the percent integrated density, the ratio of the integrated density in each ROI to the sum of integrated density in the white or gray matter in the cervical or lumbar spinal cord (please refer to “Quantification of labeled axons” in the section “Materials and Methods” for a detailed description of the method). We first compared the percentages of the normal fibers in the contralateral dorsal funiculus (cDF) and of the abnormal fibers in the ipsilateral lateral funiculus (iLF, Figure 6A). Quantitative analyses demonstrated that the CST fibers were confined to the cDF in the wild-type mice, whereas they were present more in the iLF and less in the cDF in the *Sulf1/2* DKO mice (Figures 6B,C). Next, we compared the laterality. The CST fibers projected to the gray matter on the contralateral side in the wild-type mice, whereas they projected bilaterally in the DKO mice (Figures 6D,E). Finally, we compared the dorsoventral distribution of the CST fibers. For this purpose, we divided the spinal cord into four parts along the dorsoventral axis and compared the distribution in the dorsal quarter, intermediate half, and ventral quarter (Figure 6A). This analysis showed that when the signals on both sides were added, the dorsoventral distribution within the gray matter was almost the same in the wild-type and DKO mice (Figures 6F,G).

### Topographical Organization of the Motor Area in *Sulf1/2* DKO Mice

We then wondered whether the topographical organization of the motor cortex is the same in wild-type and *Sulf1/2* DKO mice. To address this question, we performed systematic injection of BDA at different sites throughout the sensorimotor cortex (Supplementary Figure S3) and examined the projection in the cervical and lumbar spinal cord regions. Dense projection to the cervical spinal cord was found when BDA was injected at +1.2 and 0 AP, from 1.0 to 2.0 ML in both the wild-type and the DKO mice (Supplementary Figure S3). Projection to the lumbar spinal cord was found when BDA was injected at 0 AP, from 1.0 to 1.5 ML, and at −1.2 AP, from 0.5 to 1.5 ML in both the wild-type and the DKO mice (Supplementary Figure S4), indicating that the motor area projecting to the lumbar spinal cord was caudal and medial to that projecting to the cervical spinal cord, with overlap between them, which is consistent with the motor cortex map determined by electrical or optogenetic stimulation (Li and Waters, 1991; Ayling et al., 2009; Tennant et al., 2011). Thus, the overall topographical organization of the motor area of the wild-type and DKO mice was roughly the same. These data also suggest that in DKO mice, the CST fibers take two routes through the contralateral dorsal funiculus and the ipsilateral lateral funiculus regardless of the positions of their origin in the motor cortex and that they terminate at their inherent levels regardless of the route they take.

### M1-Stimulated Bilateral Responses in *Sulf1/2* DKO Mice

**FIGURE 7:**
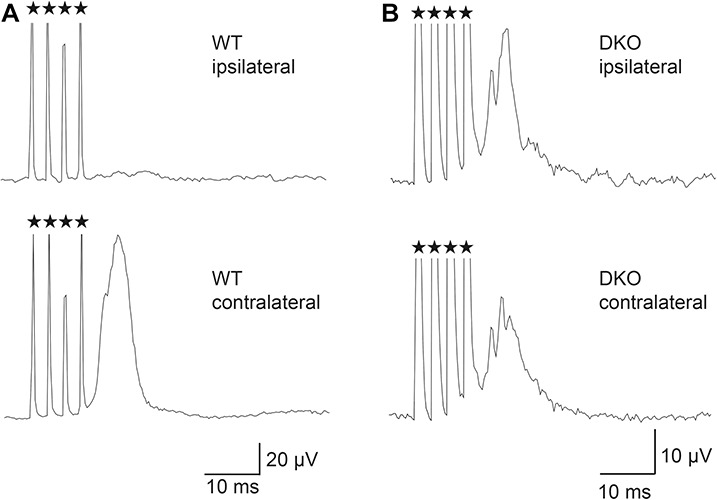
Motor-evoked potentials in the forelimb muscles. **(A,B)** Representative EMG responses in the triceps muscle in the wild-type **(A)** and DKO mice **(B)**. Responses of EMGs in bilateral triceps muscles to four square-pulses of current stimulations (200-μs duration, 3-ms intervals, every 550 ms, 50–100 μA) to the M1 cortex were recorded. The EMGs were rectified and averaged 500 times. M1 stimulation on one side evoked motor potentials only in the contralateral triceps muscles in the wild-type mice, whereas the same stimulation evoked bilateral responses in the *Sulf1/2* DKO mice. The asterisks indicate stimulation artifacts.

Our BDA tracing studies clearly demonstrated that the CST fibers projected bilaterally in the spinal cord of the *Sulf1/2* DKO mice. This observation led us to wonder whether the uncrossed fibers projecting to the ipsilateral spinal cord in the DKO mice control motor neuron activity. To test this, we measured the EMG responses after intracortical microstimulation of the M1. An anesthetized mouse was placed in a stereotaxic frame and a stimulating electrode was inserted into the M1 area. Electrical stimulation of the M1 on one side evoked contralateral forelimb movements in the wild-type mice, whereas the same stimulation evoked bilateral movements in the DKO mice. Consistent with the forelimb movements, when EMG responses were observed, activation of the EMG after four pulses of intracortical microstimulations was observed only from the contralateral muscles of the wild-type mice, whereas bilateral responses were detected in the DKO mice (Figure 7 and Supplementary Figure S5). These results show that the CST fibers are functionally connected to both the ipsilateral and the contralateral motor neurons, thereby controlling bilateral motor neuron activity in the DKO mouse.

### Deficits in Fine Motor Movements in *Sulf1/2* DKO Mice

**FIGURE 8:**
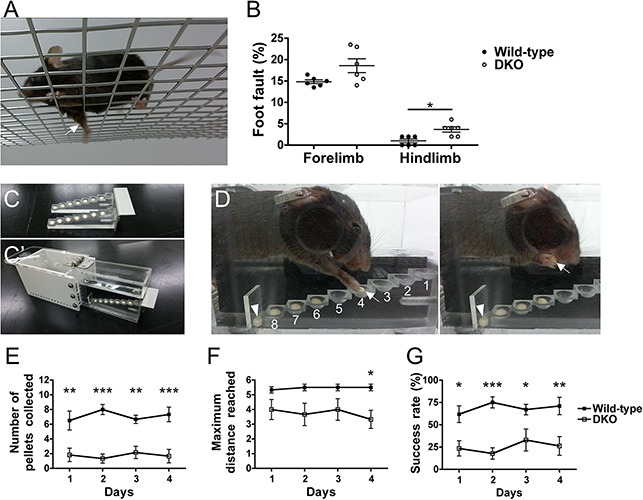
Grid-walking and staircase tests. **(A,B)** Grid-walking test. When one of the limbs fell below the grid surface during free walking on the grid (**A**, arrow), it was counted as a foot-fault error. **(B)** The foot-fault errors during the first 200 steps taken by the forelimbs and the first 50 steps taken by the hindlimbs were analyzed. The foot-fault rate was higher in the *Sulf1/2* DKO mice than in the wild-type mice: the difference was significant in the hindlimbs (3.7 ± 0.6 vs. 1.0 ± 0.4; *n* = 6; ^∗^*P* < 0.05, Mann–Whitney *U* test) but not in the forelimbs (18.6 ± 1.6 vs. 14.8 ± 0.4; *P* = 0.065). **(C–G)** Staircase test. **(C)** Double staircase separated from the chamber. Single food pellets were placed on each well in the lower seven steps on both sides. **(C′)** Staircase test apparatus. The double staircase was inserted into the space between the platform and walls. A mouse was placed into the white chamber (left) and allowed to enter the central platform (right) and retrieve pellets for 30 min. **(D)** Video recording of mouse behavior. The mouse reached, grasped, and brought a pellet to its mouth successfully (arrows). When a mouse failed to retrieve a pellet, it was moved to a different well or to the inaccessible floor (arrowheads). **(E–G)** Results of the double staircase test. The “number of pellets collected” and “success rate” were lower in the *Sulf1/2* DKO mice (*n* = 6) than in the wild-type mice (*n* = 6) throughout the test period **(E,G)**. The “maximum distance reached” indicates the deepest well reached by the mouse. Please refer to the section “Materials and Methods” for a detailed description of the analysis. Data shown are means ± SEMs. Statistical significance was calculated using two-way repeated-measures ANOVA with a Bonferroni *post hoc* test (**E–G**; ^∗^*P* < 0.05, ^∗∗^*P* < 0.01, ^∗∗∗^*P* < 0.001).

The *Sulf1/2* DKO mice appeared to behave normally: they did not show gait disturbance, ataxia, or abnormal movements. Consistent with this, the DKO mice performed normally in the rotarod and open field tests (Supplementary Figures S6A,B), suggesting that gross motor movements were normal in the DKO mice. In addition, in the hot plate test, the wild-type and DKO mice showed no significant differences in the time taken to avoid painful thermal stimuli, indicating that temperature and pain sensation was normal in the DKO mice (Supplementary Figure S6C). To examine possible deficits in fine motor movements in *Sulf1/2* DKO mice, we performed three different behavioral tests that have been used for evaluating the motor functions of normal mice and disease models (Brooks and Dunnett, 2009; Schönfeld et al., 2017). First, we performed a grid-walking test that can assess sensorimotor function and motor coordination (Starkey et al., 2005). In this test, the mice were made to walk on an elevated wire grid for 3 min and foot-fault errors were analyzed (Figure 8A). The rate for the foot fault was higher in the *Sulf1/2* DKO mice than in the wild-type mice: the difference was significant in the hindlimbs but not in the forelimbs (Figure 8B).

We next performed a double staircase test (Montoya et al., 1991; Baird et al., 2001). To this end, we used an apparatus that has a start chamber and a narrow corridor with a central platform and a double staircase (Figure 8C′). The removable staircase has eight steps on each side, and small food pellets are placed in the shallow well of each step (Figure 8C). In this apparatus, a mouse can retrieve pellets on either side using the forelimb of the same side (Figure 8D, arrows), enabling evaluation of the mouse’s ability to reach the pellets with the respective forelimbs. A food-deprived mouse was placed in the apparatus with single pellets baited on each well on both sides, and the mouse was allowed to retrieve pellets freely for 30 min over 4 days. At the end of each session, the number and place of the remaining pellets were examined. The number of pellets that the mouse ate successfully (“number of pellets collected”) was lower in the DKO mice than in the wild-type mice (Figure 8E). The “success rate,” which was calculated by dividing the “number of pellets collected” by the sum of the wells reached by the mouse on both sides (Figure 8F), was also lower in the DKO mice than in the wild-type mice throughout the test period (Figure 8G), suggesting motor deficits in forelimb movement.

**FIGURE 9:**
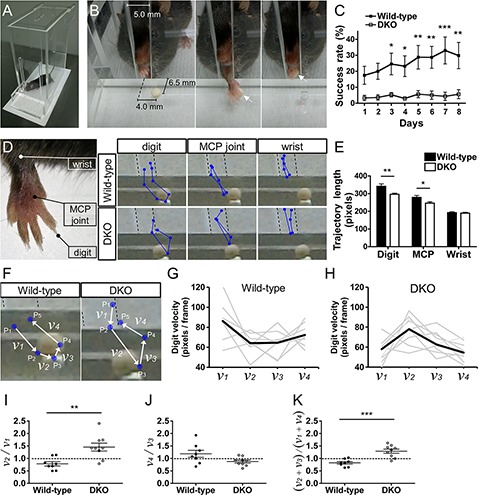
Single pellet-reaching task. **(A)** Apparatus for the single pellet-reaching task. **(B)** In a successful reach, a mouse extends a forelimb through the slit, grasps a pellet in a divot, and brings it to its mouth (arrows). **(C)** Rate of successful reaching during eight test sessions. The wild-type mice (*n* = 8) showed improvements in the success rate over time [*F*(7,49) = 3.19, *P* = 0.0072], whereas the *Sulf1/2* DKO mice (*n* = 10) showed a lower success rate throughout the test period [*F*(7,63) = 0.749, *P* = 0.63]. As a result, the success rates of the DKO mice were lower than those of the wild-type mice after the third session. **(D,E)** Trajectory analysis of the forelimb in the successful reaches. The positions of the distal tip of the second digit, second metacarpophalangeal (MCP) joint, and wrist were marked in the video (**D**, blue dots) to analyze the reaching trajectories. Representative trajectories (blue lines) in the wild-type and *Sulf1/2* DKO mice are shown. The dotted lines indicate the position of the slit. The trajectory length of the digit and MCP joint was shorter in the *Sulf1/2* DKO mice (*n* = 9) than in the wild-type mice (*n* = 8) **(E)**. **(F–K)** Velocity analysis of the forelimb in successful reaches. The positions of the distal tip of the second digit in the five consecutive frames (P_1_–P_5_) in a successful reach, in which P_3_ corresponds to the points of pellet grasping, were marked in the video, and the velocity between 2 points (*v*_1_ to *v*_4_) was measured **(F)**. In the wild-type mice, *v*_2_ and *v*_3_ were smaller than *v*_1_ and *v*_4_, respectively **(G,I–K)**. In contrast, in the *Sulf1/2* DKO mice, *v*_2_ and *v*_3_ were larger than *v*_1_ and *v*_4_, respectively **(H–K)**. Statistical significance was calculated using one-way and two-way repeated-measures ANOVA with a Bonferroni *post hoc* test in **(C,E)** and a Mann–Whitney *U* test in **(I–K)**. ^∗^*P* < 0.05, ^∗∗^*P* < 0.01, ^∗∗∗^*P* < 0.001. Data shown are means ± SEMs.

We then performed a single pellet-reaching test (Farr and Whishaw, 2002; Chen et al., 2014). In this test, the mice were trained to use a forelimb to retrieve a small pellet through a narrow slit of a clear box (Figures 9A,B). First, a food-deprived mouse was trained to retrieve pellets from the center slit until the mouse performed 20 reaching attempts within 20 min. Because the mouse used both forelimbs, the preferred limb was determined. In the subsequent test sessions (one session per day, for 8 days), a single pellet was placed in a divot at the fixed position (Figure 9B) to force the mouse to retrieve the pellet with the preferred forelimb. The rate at which the mouse successfully grasped a pellet and brought it into its mouth was higher in the wild-type mice than in the DKO mice (Figure 9C), indicating impaired performance in pellet retrieval in the DKO mice. The success rate became higher during the test sessions in the wild-type mice but not in the DKO mice (Figure 9C and Supplementary Figure S7).

To examine why the DKO mice were less successful at reaching the pellets, we performed kinematic measures of the trajectory and velocity of the forelimb movement (Whishaw, 1996). The positions of the distal tip of the second digit, second metacarpophalangeal (MCP) joint, and wrist were marked separately in single frames and each trajectory was examined (Figure 9D). As shown in Figure 9E, the trajectories of the digit and MCP joint, but not of the wrist, in the successful reaches were shorter in the DKO mice than in the wild-type mice. In addition, the speed of the digit near the pellet was slowed down in the wild-type mice but accelerated in the DKO mice (Figures 9F–K). This analysis revealed that DKO mice use different goal-directed action strategies even in successful reaches.

## Discussion

In this study, by taking advantage of the survival into adulthood of *Sulf1/2* DKO mice on a mixed genetic background, we demonstrated several aspects of the CST defects in the adult brain. First, the abnormal dorsal extension of the CST fibers toward the superior colliculus, which was observed in DKO embryos (Okada et al., 2017), persisted in the adult brain. Second, at the pyramidal decussation, some fibers located close to the midline crossed the midline, whereas others located more laterally did not. Third, in the spinal cord, the crossed fibers descended in the contralateral dorsal funiculus and entered the contralateral gray matter, whereas the uncrossed fibers descended in the lateral funiculus on the ipsilateral side and entered the ipsilateral gray matter. These results showed that the CST fibers of *Sulf1/2* DKO mice project bilaterally in the spinal cord. Consistently, electric stimulation of M1 neurons on 1 side evoked bilateral EMG responses in the DKO mice. We also demonstrated impaired performance of the DKO mice in behavioral tests used for evaluating motor functions, suggesting deficits in their motor movement.

### Pyramidal Decussation Defects

Various CST defects at the pyramidal decussation were reported in mutant mice (Canty and Murphy, 2008; Leyva-Díaz and López-Bendito, 2013; Welniarz et al., 2017a). These mutant mice include mutants for Semaphorin 6A (*Sema6a*) and its receptors Plexin A3 (*Plxna3*)/Plexin A4 (*Plxna4;* Faulkner et al., 2008; Rünker et al., 2008); Netrin receptors DCC (*Kanga* mutant expressing a truncated DCC protein [*Dcc*^*kanga*^]) and UNC5C (*rostral cerebellar malformation* mutant, *Unc5c*^*rcm*^; Finger et al., 2002); and cell adhesion molecules NCAM (*Ncam1*; Rolf et al., 2002) and L1 (*L1cam*; Dahme et al., 1997; Cohen et al., 1998).

In the *Sema6a*, *Plxna3*, or *Plxna4* KO mice and the *Plxna3/Plxna4* DKO mice, a part of the CST fibers deviated from the midline and ran down the ventrolateral position of the medulla, whereas those near the midline decussated normally (Faulkner et al., 2008; Rünker et al., 2008). The authors of those studies argued that the phenotype was caused by the loss of the constraint of the CST fibers to the midline, which is normally brought about by the repulsive signal of Sema6A in the inferior olive (Faulkner et al., 2008; Rünker et al., 2008). The CST abnormalities of these KO mice seem similar to those of the *Sulf1/2* DKO mice. However, the *Sema6a* KO mice showed aberrant circumferential projection of the CST fibers in the medulla (Okada et al., 2019), which was never observed in the *Sulf1/2* DKO mice, indicating that the molecular mechanisms causing the decussation defects differ for *Sema6a-Plxna3/a4* and *Sulf1/2* mutant mice.

In the *Dcc*^*kanga*^ mutant mice, the CST fibers spread into the medial and lateral bundles just before the pyramidal decussation and neither of them crossed the midline (Finger et al., 2002; Welniarz et al., 2017b). The medial bundle extended ipsilaterally in the ventral funiculus, whereas the lateral bundle extended ipsilaterally in the ventral portion of the lateral funiculus (Finger et al., 2002). Thus, the CST defects in the *Dcc*^*kanga*^ mutant mice seem to differ from those in the *Sulf1/2* DKO mice. In the *Unc5c*^*rcm*^ mutant mice, the CST fibers formed two distinct bundles at the pyramidal decussation: one bundle in the normal position near the midline and another located more laterally. Whilst the fibers of the former crossed the midline normally, most entered the dorsal gray matter adjacent to the dorsal funiculus instead of entering into the dorsal funiculus (Finger et al., 2002). Thus, the properties of the crossed fibers differ from those in the *Sulf1/2* DKO mice. By contrast, the fibers of the laterally located bundle descended ipsilaterally in the outermost region of the lateral funiculus of the spinal cord (Finger et al., 2002). Therefore, the decussation error phenotype in the *Unc5c* mutant mice is similar to that in the *Sulf1/2* DKO mice. In this regard, it is interesting that different types of HS selective for DCC and UNC-5 are assumed to lead to the formation of the DCC/DCC and DCC/UNC-5 complex with Netrin-1, thereby causing axonal attraction and repulsion, respectively (Finci et al., 2014). Sulf1/2-mediated modification of HS may affect HS-dependent formation of the netrin receptor complex and consequent netrin-mediated responses.

In the *L1cam* and *Ncam1* KO mice, a significant portion of the CST fibers failed to cross the midline and entered the ipsilateral dorsal funiculus (Cohen et al., 1998; Rolf et al., 2002). In addition, in the *Ncam1* KO mice, the CST fibers frequently extended laterally instead of growing dorsally (Rolf et al., 2002). In the *L1cam* KO mice, the CST fibers were reduced in number and not observed caudal to the cervical spinal cord (Dahme et al., 1997; Cohen et al., 1998). These phenotypes differ largely from those observed in the *Sulf1/2* DKO mice.

These comparisons revealed that none of the mice with loss-of-function mutations in one of these genes showed the same CST phenotype as that of the *Sulf1/2* DKO mice. It is possible that the gain-of-function mutations in these genes are related to the phenotype of the DKO mice or that the combination of mutations in more than one gene causes the phenotype.

In the *Sulf1/2* DKO mice, the CST fibers within approximately 700 μm of the midline decussated almost normally, whereas the fibers outside this border did not, suggesting that the midline-crossing signals may operate within this range. Viewed from this standpoint, it is likely that the location of the CST fibers in the medulla after their return from the aberrant detour in the midbrain may determine whether they cross the midline. Lateral positioning of the CST fibers may be influenced by Slit-Robo signaling, as proposed in mutant *Drosophila* and mouse studies (Rajagopalan et al., 2000; Simpson et al., 2000; Farmer et al., 2008; Jaworski et al., 2010).

### Descending CST Fibers in the Lateral Funiculus of the Spinal Cord

The CST is a major descending pathway that appeared in mammals evolutionarily. Although the anatomy of the CST is similar across species, the location of its fibers in the spinal cord is different from one species to another (Lemon, 2008; Welniarz et al., 2017a). In rodents, the crossed fibers are located in the ventral portion of the dorsal funiculus, whereas a small number of uncrossed fibers are present in the ventral funiculus (Brösamle and Schwab, 1997). Despite the different locations, the crossed and uncrossed fibers originate from the same cortical areas (Brösamle and Schwab, 1997). In primates, by contrast, the crossed fibers are located in the dorsolateral funiculus, whereas the uncrossed fibers are located in the ventral and dorsolateral funiculi (Welniarz et al., 2017a). In *Sulf1/2* DKO mice and in *Dcc* and *Unc5c* mutants, the uncrossed fibers descend in the lateral funiculus, suggesting that it provides a permissive substrate for CST axon elongation beyond species. It will thus be interesting to investigate the molecular mechanisms that determine the trajectory choice, especially the genetic elements that influence species differences, as shown in a previous study that revealed the mechanism underlying manual dexterity (Gu et al., 2017). In addition, our finding that the CST fibers of the *Sulf1/2* DKO mice terminate in their inherent levels irrespective of the route they take suggests the presence of mechanisms that stop the projection of the growing axons at the appropriate levels of the spinal cord.

### Bilateral Projection of the CST Fibers in the Spinal Cord

In rodents, the majority of CST fibers cross the midline at the pyramidal decussation and project to the contralateral spinal cord (Welniarz et al., 2017a). In *Sulf1/2* DKO mice, in addition to the normal, contralateral projection of the crossed fibers, the uncrossed fibers enter the ipsilateral spinal cord, resulting in bilateral projection. Our EMG studies showed that microstimulation of the M1 on 1 side evoked comparable responses in the bilateral forelimb muscles, indicating that the uncrossed fibers also form functional synapses and control the ipsilateral motor activity.

Bilateral CST projection was also observed in other mutant mice that lack ephrin B3 (*Efnb3*), EphA4 (*Epha4*), or α-chimerin (*Chn1*), although the location of the abnormal midline crossing differs between these mice and *Sulf1/2* DKO mice. In the above mutants, the CST fibers cross the midline at the pyramidal decussation and descend in the contralateral dorsal funiculus normally, but a considerable amount of the fibers cross the midline again after entering the gray matter, resulting in bilateral projection (Dottori et al., 1998; Kullander et al., 2001a, b; Yokoyama et al., 2001; Iwasato et al., 2007). In addition to the misprojection of the CST fibers, axons of the interneurons in the spinal cord cross the midline abnormally as a result of the loss of the midline barrier signal by ephrin B3. Interestingly, these mutant mice show a hopping gait phenotype, which was thought to be caused by the inability to perform left-right alternate movement during locomotion (Kullander et al., 2003; Borgius et al., 2014; Serradj et al., 2014; Katori et al., 2017). Spinal cord-specific deletion of *Epha4* or *Chn1* led to a hopping gait, whereas forebrain-specific disruption of the same genes did not lead to the phenotype (Borgius et al., 2014; Katori et al., 2017), suggesting that a hopping gait is caused by abnormal midline crossing of interneurons in the spinal cord. Given that *Sulf1/2* DKO mice did not show a hopping gait, they did not have deficits in the locomotor circuit, although a few CST fibers crossed the midline in the spinal cord in those mice.

Interestingly, neurological conditions similar to the anatomical and functional CST abnormalities found in the *Sulf1/2* DKO mice are observed in patients with congenital mirror movement (CMM). CMM is a rare genetic disorder that is characterized by involuntary movements on one side of the body induced by intentional movements on the opposite side (Galléa et al., 2011; Cox et al., 2012; Welniarz et al., 2017a) as the result of mutations in the *DCC*, *RAD51*, and *NTN1* (netrin-1) genes (Srour et al., 2010; Depienne et al., 2012; Méneret et al., 2017). In some CMM patients, the proportion of the uncrossed CST fibers at the pyramidal decussation was increased and unilateral M1 stimulation elicited bilateral responses (Méneret et al., 2017; Welniarz et al., 2017b). Therefore, the patterns of the anatomical and physiological deficits are almost the same as those in the *Sulf1/2* DKO mice, although these mice did not show apparent mirror movement in their cage or in our behavioral tests. However, forebrain-specific *EphA4* KO mice, which did not show a hopping gait in normal stereotypical locomotion despite the existence of abnormal midline crossing of the CST fibers in the spinal cord, showed abnormal synchronization of left and right forelimb movement in adaptive locomotion over obstacles (Serradj et al., 2014). Thus, it would be interesting to examine whether *Sulf1/2* DKO mice show synchronized movement in the same behavioral test. It may be useful to elucidate the contribution of midline-crossing abnormality to mirror movement and to address the question whether midline-crossing errors at the pyramidal decussation and in the spinal cord have different consequences for motor control in left-right synchronization.

### Impaired Motor Movement

The CST plays an important role in cortical control of spinal motor neuron activity (Lemon, 2008; Welniarz et al., 2017a). Because it is a major pathway for voluntary movement, its dysfunction leads to motor impairment. In mice, lesions of the motor cortex, surgical dissection of the pyramidal tract, or optogenetic silencing of the corticospinal neurons led to motor deficits (Baird et al., 2001; Farr and Whishaw, 2002; Starkey et al., 2005; Ueno et al., 2018). The *Sulf1/2* DKO mice showed impaired performance in the staircase test and single pellet-reaching tests, although their gross movements and locomotion appeared to be normal, indicating deficits in fine motor movement. In the DKO mice, in the single pellet-reaching test, the trajectories of the distal component of the forelimb and speed control near the target were different from those in the wild-type mice, indicating that the DKO mice use different goal-directed action strategies. We examined the correlation between the parameters obtained from the kinematic measurement in the single pellet-reaching test (performance, trajectory length, and speed) and the anatomical defects of the CST (signal intensity of the PKCγ staining in the contralateral/ipsilateral and dorsal/lateral funiculi) in the DKO mice, but we could not find any significant correlation between them. Analysis of the synaptic connections in the spinal cord may be necessary, although it appears to be difficult to attribute some aspect of the behavioral deficits to simple anatomical phenotypes.

In rodents, CST axons originate from the motor, somatosensory, parietal, cingulate, visual, and prefrontal regions and mediate many different functions (Lemon, 2008; Welniarz et al., 2017a). Therefore, movement impairment in *Sulf1/2* DKO mice may be caused by deficits of CST function that include descending control of afferent inputs and gating and gain control of the spinal reflex in addition to excitation of motor neurons (Lemon, 2008; Welniarz et al., 2017a). Involvement of the CST fibers originating from the somatosensory cortex or other areas that affect their performance (Lemon, 2008; Wang et al., 2017; Ueno et al., 2018) should also be considered. Furthermore, it is also important to note that sensory feedback to the CST plays a critical role in controlling movement (Seki et al., 2003; Bui et al., 2013; Bourane et al., 2015). Although the sensory function assessed by the hot plate test was normal in the DKO mice, the possibility cannot be excluded that the sensory deficits due to the abnormality of the CST fiber projections from the sensory cortex affected the performance in the behavioral tests. To understand the mechanism of impaired motor movements in *Sulf1/2* DKO mice, it will be important to determine whether motor deficits are caused by reduction in the number of normal crossed fibers or by interference in the normal functions of the crossed fibers by the presence of abnormal ipsilateral fibers.

## Data Availability Statement

The datasets generated for this study are available on request to the corresponding author.

## Ethics Statement

The animal study was reviewed and approved by the Animal Care and Use Committee of the University of Tsukuba.

## Author Contributions

SA, TO, KK-M, and MM designed the research and performed the experiments. SA, MA, and KK-M performed the rotarod and open field tests. TD, TK, and MM performed the EGM recording. SA, TO, and TD analyzed the data. AT supervised SA and evaluated the clinical implications. SA, TO, KK-M, and MM wrote the manuscript. All the authors read and approved the final manuscript.

## Conflict of Interest

The authors declare that the research was conducted in the absence of any commercial or financial relationships that could be construed as a potential conflict of interest.

## Footnotes

1 https://imagej.nih.gov/ij/

2 https://imagescience.org/meijering/software/mtrackj/

## Abbreviations

ABC: avidin-biotin-peroxidase complex
AP: anterior-posterior
BDA: biotinylated dextran amine
CMM: congenital mirror movement
CST: corticospinal tract
DAB: 3,3′-diaminobenzidine
DF: dorsal funiculus
DKO: double knockout
DMSO: dimethyl sulfoxide
DV: dorsal-ventral
EMG: electromyogram
HS: heparan sulfate
i.p.: intraperitoneal
KO: knockout
LF: lateral funiculus
M1: primary motor cortex
ML: medial-lateral
PBS: phosphate-buffered saline
PBT: PBS with 0.1% Tween-20
PFA: paraformaldehyde
PKC γ: protein kinase C gamma
ROI: region of interest
Sulf1: Sulfatase 1
TBS: Tris-buffered saline
TBST: Tris-buffered saline supplemented with 0.1% Tween-20
TSA: tyramide signal amplification.
