# Supplementary material for: Abnormal Pyramidal Decussation and Bilateral Projection of the Corticospinal Tract Axons in Mice Lacking the Heparan Sulfate Endosulfatases, Sulf1 and Sulf2
Source: Front Mol Neurosci. 2020 Jan 21;12:333. doi: 10.3389/fnmol.2019.00333 (PMC6985096; doi:10.3389/fnmol.2019.00333)
Supplement: Supplementary file 1 [file Data_Sheet_1.docx]

**Supplementary material**

**Abnormal pyramidal decussation and bilateral projection of the corticospinal tract axons in mice lacking the heparan sulfate endosulfatases, Sulf1 and Sulf2**

**Satoshi Aizawa^1,2,3^, Takuya Okada^1,2^, Kazuko Keino-Masu^1,2^, Tri Huu Doan^1,4^, Tadachika Koganezawa^1,4,5^, Masahiro Akiyama^6^, Akira Tamaoka^1,3^, Masayuki Masu^1,2*^**

^1^Graduate School of Comprehensive Human Sciences, University of Tsukuba, Tsukuba, Ibaraki, Japan

^2^Department of Molecular Neurobiology, Division of Biomedical Science, Faculty of Medicine, University of Tsukuba, Tsukuba, Ibaraki, Japan

^3^Department of Neurology, Division of Clinical Medicine, Faculty of Medicine, University of Tsukuba, Tsukuba, Ibaraki, Japan

^4^Department of Physiology, Division of Biomedical Science, Faculty of Medicine, University of Tsukuba, Tsukuba, Ibaraki, Japan

^5^Transborder Medical Research Center, Faculty of Medicine, University of Tsukuba, Tsukuba, Ibaraki, Japan

^6^Environmental Biology Laboratory, Faculty of Medicine, University of Tsukuba, Tsukuba, Ibaraki, Japan

***Correspondence:**

Masayuki Masu, Department of Molecular Neurobiology, Division of Biomedical Science, Faculty of Medicine, University of Tsukuba, 1-1-1 Tennodai, Tsukuba, Ibaraki 305-8575, Japan

Tel: +81-29-853-3249

Fax: +81-29-853-3498

mmasu@md.tsukuba.ac.jp

**Supplementary figures**

**Supplementary Figure S1.** Variation in the pyramidal decussation defects of *Sulf1/2* DKO mice.

**(A–F)** 3D reconstructed images of the pyramidal decussation in 2 *Sulf1/2* DKO mice. Lateral (**A, D**), frontal (**B, E**), and ventral (**C, F**) views are shown. The yellow and white dotted lines indicate the midline and contours of the brain, respectively. The white and yellow brackets indicate the medial and lateral bundles, respectively. The anterior-posterior (A-P) and dorsal-ventral (D-V) body axes are shown. df, dorsal funiculus; py, pyramidal tract; pyx, pyramidal decussation. The scale bar indicates 1.0 mm. Please see Figure **2F–H** and **2J–L**.

**Supplementary Figure S2.** Midline crossing of the CST fibers in the *Sulf1/2* DKO spinal cord.

**(A–C)** Transverse sections of the cervical spinal cords of *Sulf1/2* DKO mice. The CST fibers were labeled by stereotaxic injection of BDA into the motor cortex (coordinate AP +1.2, ML +2.0, DV +0.7 in [**A**] and AP ±0, ML +1.5, DV +0.7 in [**B–C**], in mm). Some CST fibers crossed the midline abnormally in *Sulf1/2* DKO mice (**A’–C’**; arrowheads). The dashed lines in (**A–C**) delineate the borders of the dorsal funiculus and ipsilateral lateral funiculus. The solid lines in (**A–C**) and the dashed lines in (**A’–C’**) indicate the midline. (**A’–C’**) show the magnified images of the boxed regions in (**A–C**). cc, central canal. The scale bar indicates 500 µm (**A–C**) and 100 µm (**A’–C’**).

**Supplementary Figure S3.** Relationship between BDA injection sites and the distribution of the labeled CST fibers in the cervical spinal cord.

**(A–F)** Representative images of the transverse sections of the cervical spinal cords of the control (**A–C**) and *Sulf1/2* DKO (**D–F**) mice that underwent BDA injection at the positions indicated in the boxes at the top (ML axis) and left (AP axis) of the images. In addition to the wild-type mice (**A_3_, A_5_**, **B_2_, B_4_**, **C_2_,** **C_4_**), single *Sulf1* KO (**A_2_**, **A_4_**, **B_1_, B_3_, B_5_**, **C_5_**) or *Sulf2* KO (**A_1_**, **C_1_**, **C_3_**) mice were used as the controls because they have no CST defects. The BDA injection sites are illustrated as white circles in the upper panel. Stereotaxic coordinates (in mm) are AP +1.2, ±0, and -1.2; ML 0.5, 1.0, 1.5, 2.0 2.5 and 3.0; DV 0.7. The scale bar indicates 500 µm.

**Supplementary Figure S4.** Relationship between BDA injection sites and the distribution of the labeled CST fibers in the lumbar spinal cord.

**(A–F)** Representative images of the transverse sections of the lumbar spinal cords of the control (**A–C**) and *Sulf1/2* DKO mice (**D–F**) that received BDA injection at the position indicated in the boxes at the top (ML axis) and left (AP axis) of the images. The mice used are the same as those shown in Supplementary figure **S3**. The scale bar indicates 500 µm.

**Supplementary Figure S5.** Motor-evoked potentials in the forelimb muscles.

**(A–B)** Representative EMG responses in the biceps and triceps muscles in the wild-type (**A**) and DKO (**B**) mice. The EMG responses in the bilateral biceps and triceps muscles to 4 square-pulses of current stimulations (200-µs duration, 3-ms intervals, every 550 ms, 50–100 µA) to the right or left M1 were recorded. The EMGs were rectified and averaged 500 times. M1 stimulation on 1 side evoked motor potentials only in the contralateral muscles in the wild-type mice, whereas the same stimulation evoked bilateral responses in the *Sulf1/2* DKO mice. The asterisks indicate stimulation artifacts.

**Supplementary Figure S6.** Open field, rotarod, and hot plate tests.

**(A)** Open field test. The total distance travelled during 30 min in a square arena (500 × 500 mm, wall height of 400 mm) is shown. No significant difference was found between the wild-type and *Sulf1/2* DKO mice (n = 5 for each, P = 0.245). **(B)** Rotarod test. The length of time a mouse remained on a rotating rod with accelerating speed (4–40 rpm) over 5 min is shown. No significant difference was found between the wild-type and *Sulf1/2* DKO mice (n = 5 for each, P = 0.111). **(C)** Hot plate test. Latency to jump, paw-shaking, or paw-licking on the hot plate (55°C) is shown. No significant difference was found between the wild-type and *Sulf1/2* DKO mice (n = 5 for each, 14.0 ± 1.4 vs 11.5 ± 3.1; P = 0.54). The data shown are means ± SEMs. Statistical significance was calculated using 2-way repeated measures ANOVA with a Bonferroni post hoc test in (**A–B**) and the Mann-Whitney U test in (**C**).

**Supplementary Figure S7.** Impairment of reaching attempts of *Sulf1/2* DKO mice in a single pellet-reaching test.

**(A)** Criteria for judging performance. The outcome of the reaching attempts in a single pellet-reaching test was classified into 4 categories: failure, loss, drop, and success, according to the decision tree. “Failure” means that a mouse failed to grasp the pellet. “Loss” means that a mouse grasped the pellet but lost it before passing its forelimb through the slit of the box. “Drop” means that a mouse passed its forelimb through the slit while holding the pellet but dropped it before conveying it to its mouth. “Success” means that a mouse brought the pellet to its mouth successfully. **(B)** Average rates for failure, loss, drop, and success per total attempts in wild-type (n = 8) and *Sulf1/2* DKO mice (n = 10). The data shown are means ± SEMs. Statistical significance was calculated using 2-way ANOVA with a Bonferroni post hoc test (***P < 0.001). **(C)** Average rates for failure, loss, drop, and success during 8 test days in individual mice. The mice are ordered in ascending order of the success rate in the groups of the wild-type or *Sulf1/2* DKO mice. **(D–E)** Average rates for failure, loss, drop, and success on the first to eighth days of the test period in the wild-type (**D**) and *Sulf1/2* DKO mice (**E**). The success rate increased with increasing test days in the wild-type mice [F(7, 49) = 3.19, P = 0.0072] but not in the *Sulf1/2* DKO mice [F(7, 63) = 0.749, P = 0.63]. Statistical significance was calculated using 1-way repeated measures ANOVA.

**Supplementary Table S1.** Summary of BDA tracing of CST fibers.

Information about all the mice analyzed and their phenotypes is summarized.
