# Supplementary figures and images for: Abnormal Pyramidal Decussation and Bilateral Projection of the Corticospinal Tract Axons in Mice Lacking the Heparan Sulfate Endosulfatases, Sulf1 and Sulf2
Source: Front Mol Neurosci. 2020 Jan 21;12:333. doi: 10.3389/fnmol.2019.00333 (PMC6985096; doi:10.3389/fnmol.2019.00333)

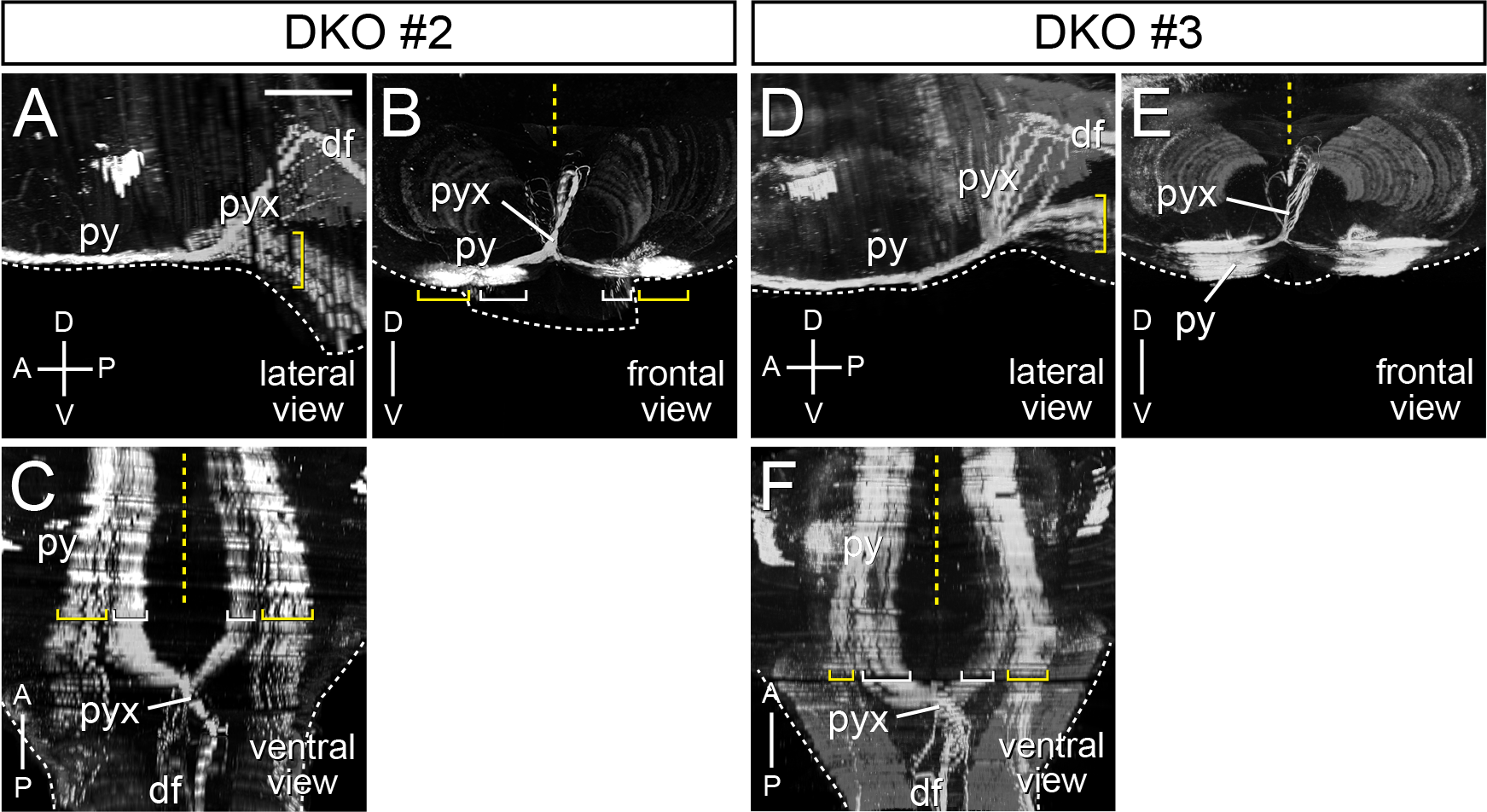

Supplement: Supplementary file 2 [file Image_1.TIFF]

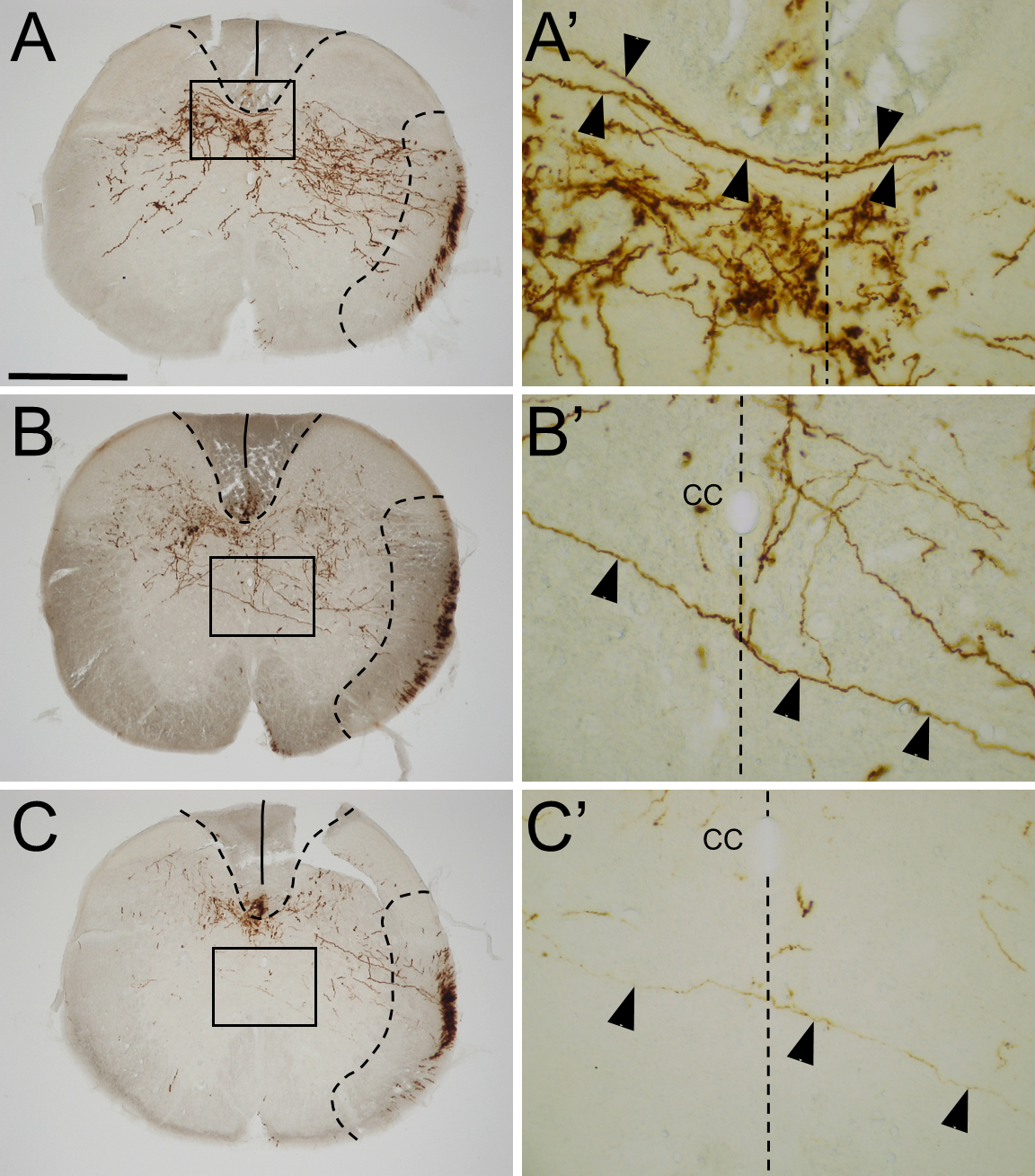

Supplement: Supplementary file 3 [file Image_2.TIFF]

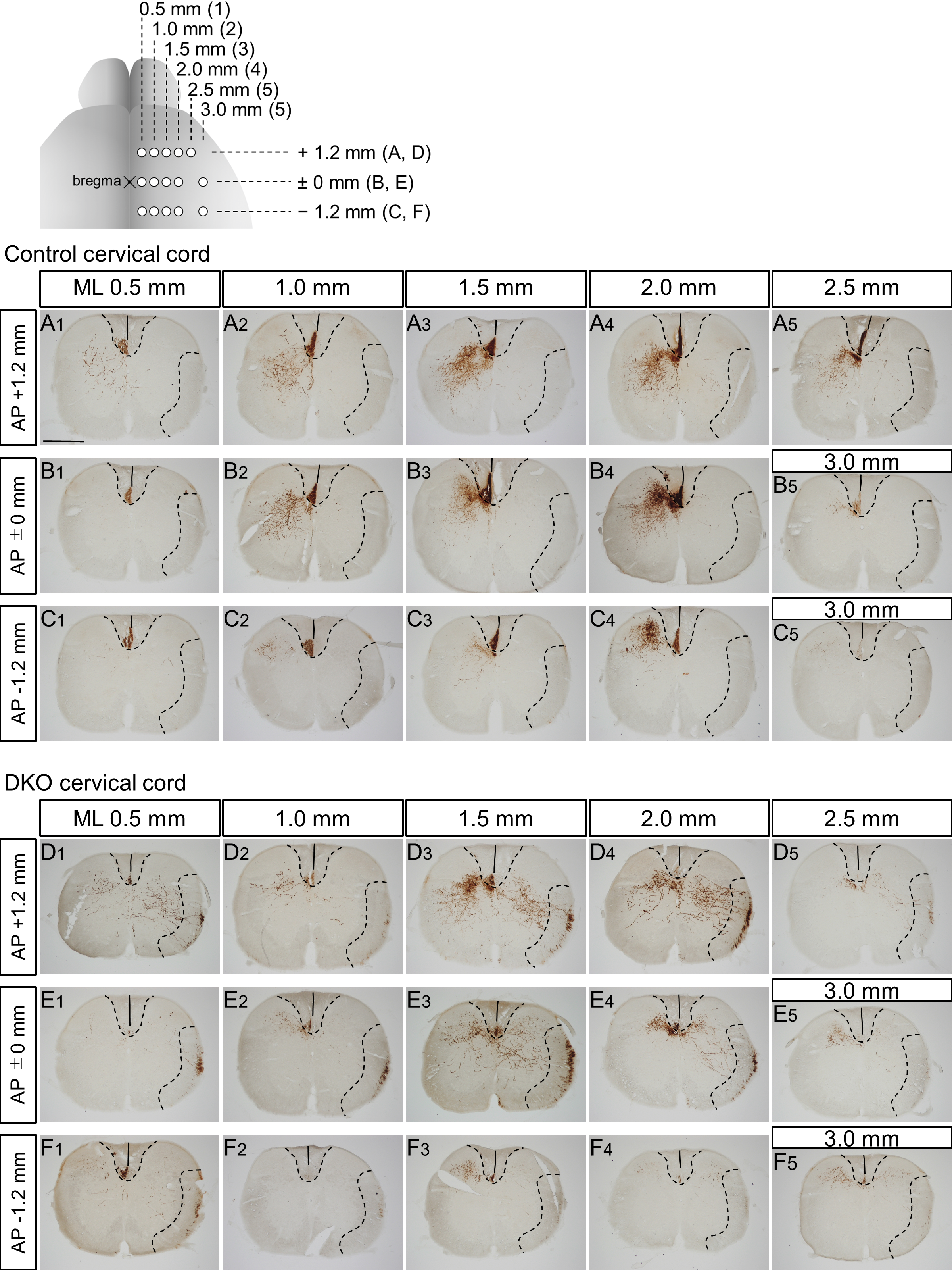

Supplement: Supplementary file 4 [file Image_3.TIFF]

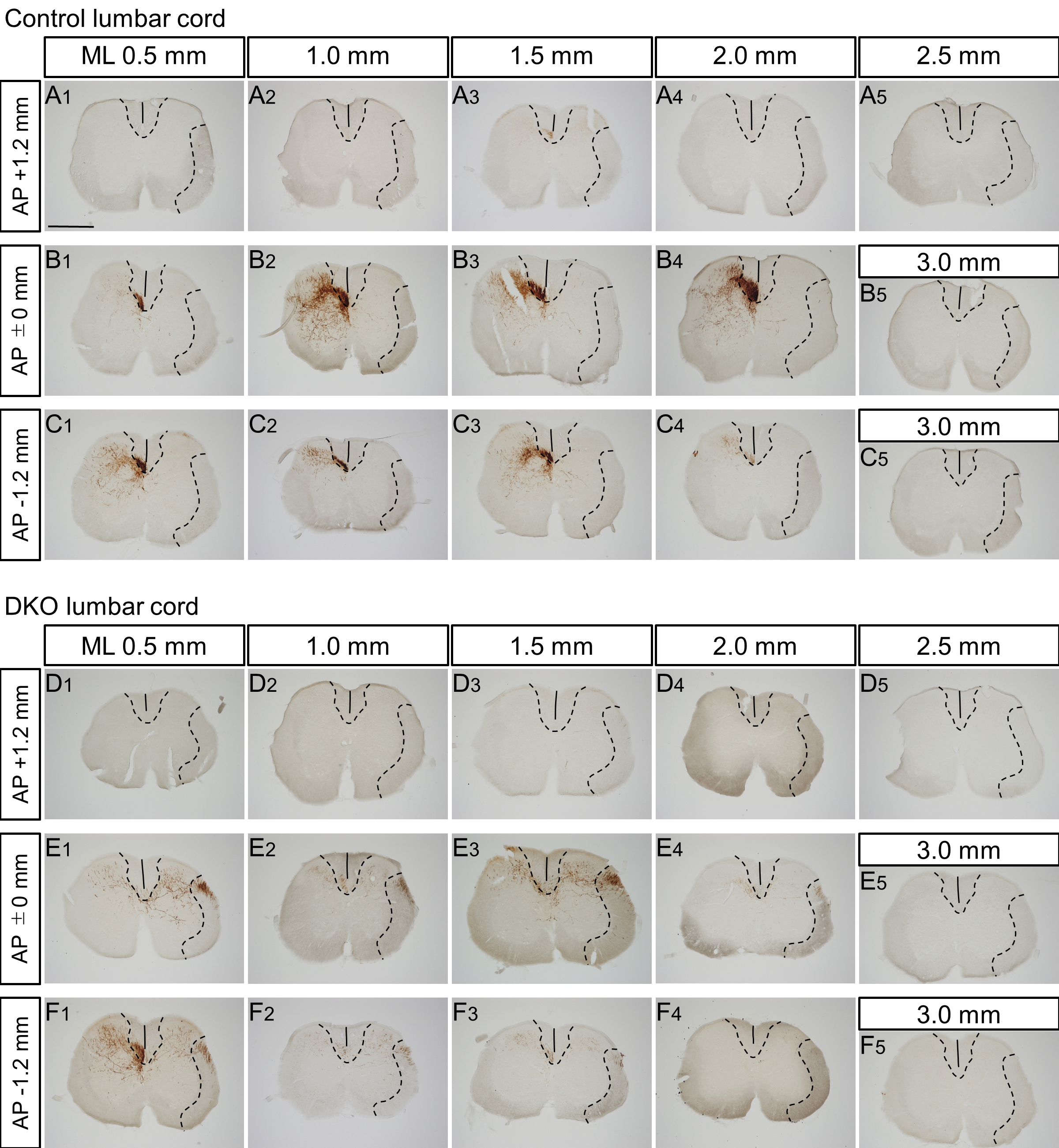

Supplement: Supplementary file 5 [file Image_4.TIFF]

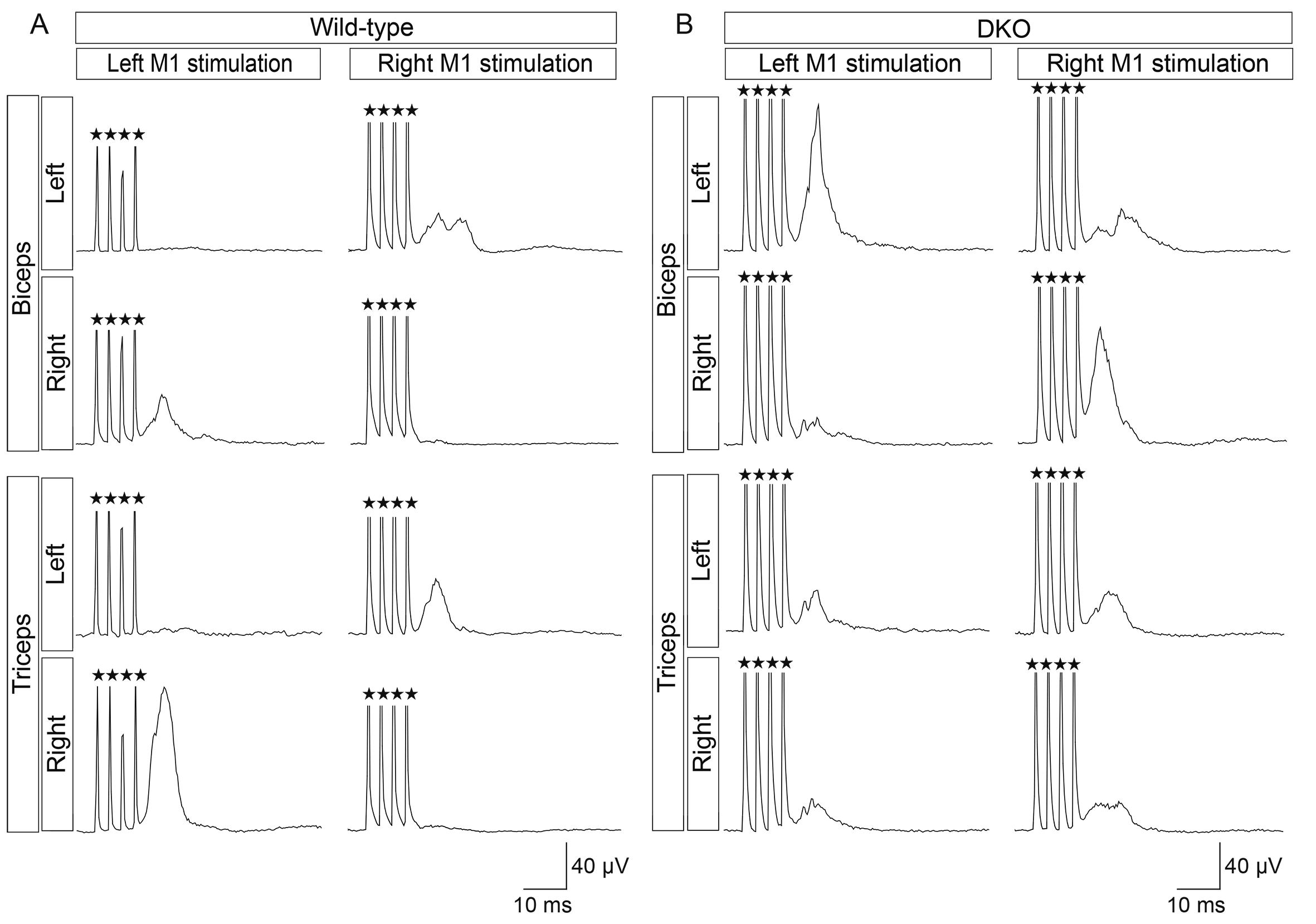

Supplement: Supplementary file 6 [file Image_5.TIFF]

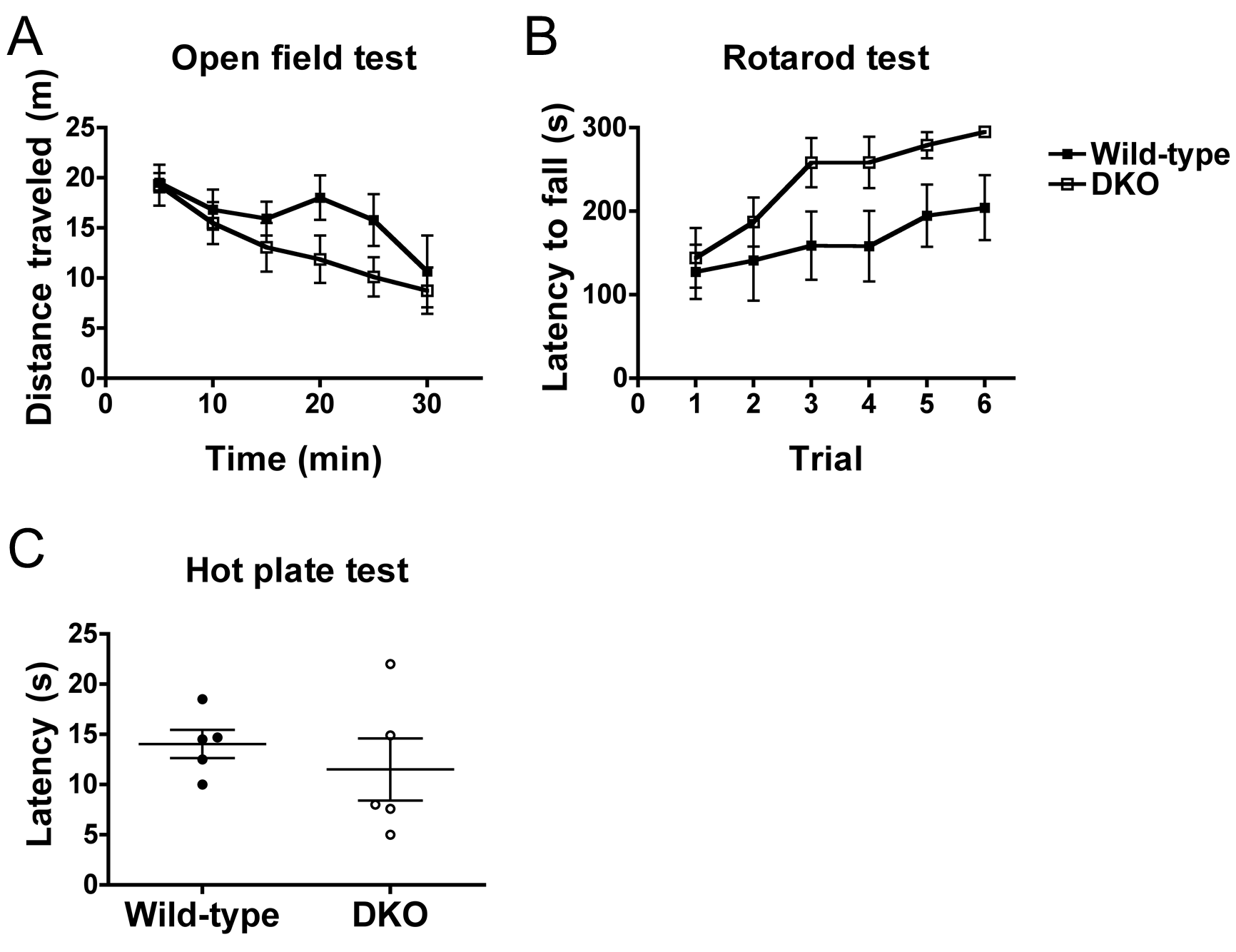

Supplement: Supplementary file 7 [file Image_6.TIFF]

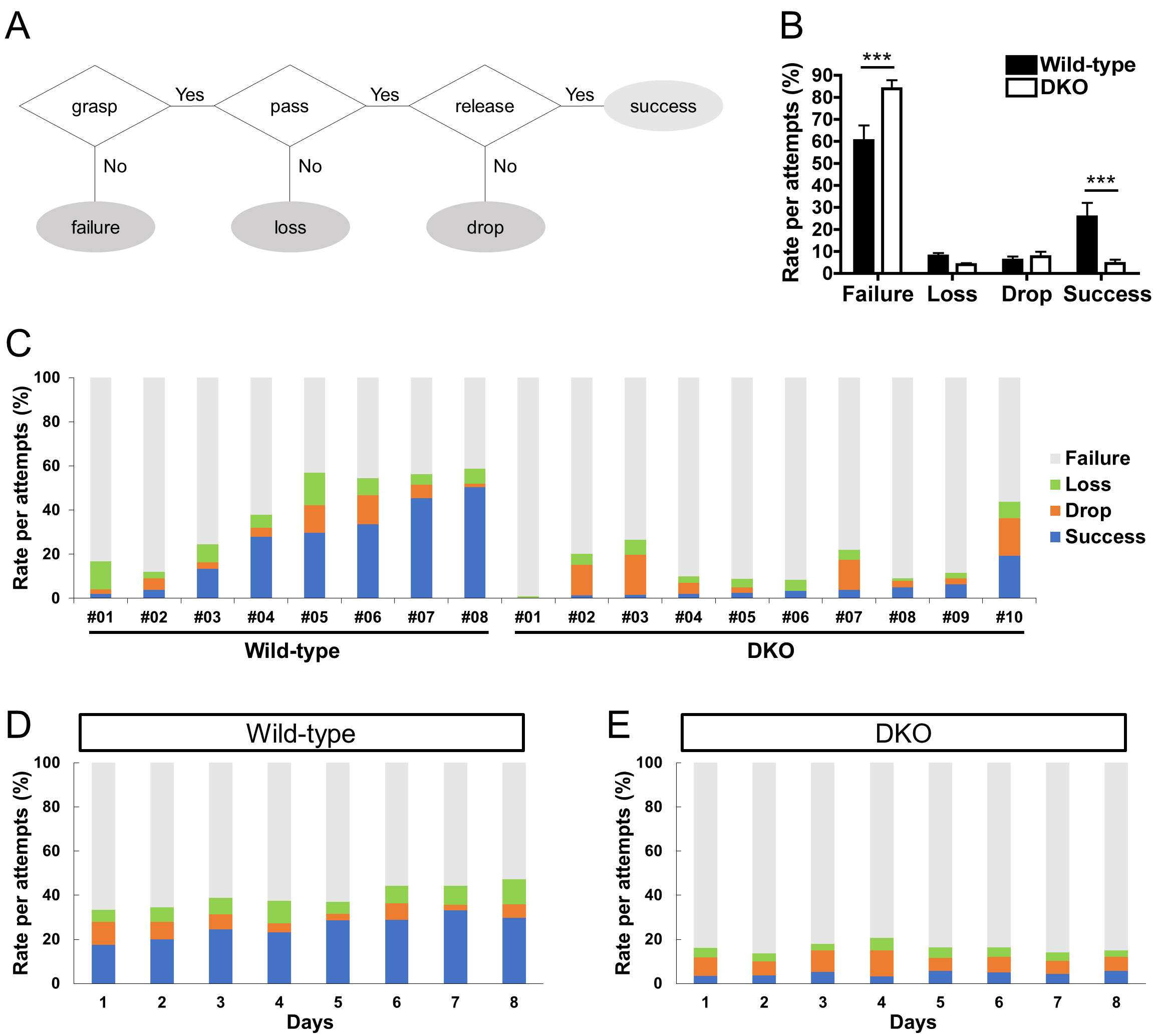

Supplement: Supplementary file 8 [file Image_7.TIFF]
